# Two-photon excited deep-red and near-infrared emissive organic co-crystals

**DOI:** 10.1038/s41467-020-18431-7

**Published:** 2020-09-15

**Authors:** Yu Wang, Huang Wu, Penghao Li, Su Chen, Leighton O. Jones, Martín A. Mosquera, Long Zhang, Kang Cai, Hongliang Chen, Xiao-Yang Chen, Charlotte L. Stern, Michael R. Wasielewski, Mark A. Ratner, George C. Schatz, J. Fraser Stoddart

**Affiliations:** 1 Department of Chemistry, Northwestern University, 2145 Sheridan Road, Evanston, IL 60208 USA; 2 School of Chemistry, University of New South Wales, Sydney, NSW 2052 Australia; 3 Institute for Molecular Design and Synthesis, Tianjin University, 92 Weijin Road, Nankai District, Tianjin 300072 P.R. China

**Keywords:** Optical materials, Crystal engineering, Self-assembly

## Abstract

Two-photon excited near-infrared fluorescence materials have garnered considerable attention because of their superior optical penetration, higher spatial resolution, and lower optical scattering compared with other optical materials. Herein, a convenient and efficient supramolecular approach is used to synthesize a two-photon excited near-infrared emissive co-crystalline material. A naphthalenediimide-based triangular macrocycle and coronene form selectively two co-crystals. The triangle-shaped co-crystal emits deep-red fluorescence, while the quadrangle-shaped co-crystal displays deep-red and near-infrared emission centered on 668 nm, which represents a 162 nm red-shift compared with its precursors. Benefiting from intermolecular charge transfer interactions, the two co-crystals possess higher calculated two-photon absorption cross-sections than those of their individual constituents. Their two-photon absorption bands reach into the NIR-II region of the electromagnetic spectrum. The quadrangle-shaped co-crystal constitutes a unique material that exhibits two-photon absorption and near-infrared emission simultaneously. This co-crystallization strategy holds considerable promise for the future design and synthesis of more advanced optical materials.

## Abstract (web summary)

Two-photon excited near-infrared fluorescence materials have garnered considerable attention because of their superior optical properties compared with other optical materials. Here, the authors use a convenient and efficient supramolecular approach to synthesize a two-photon excited near-infrared emissive co-crystalline material.

## Introduction

Organic near-infrared (NIR) absorbing and emitting materials have found applications in fields as broad as bio-imaging^1–3^, photothermal therapy^4–6^, drug release^7^, night-vision technologies, and advanced optoelectronics^8,9^, for the simple reason that NIR light exhibits superior optical penetration, lesser photodamage, and lower optical scattering compared to visible light^10–12^. In order to fabricate NIR absorbing or emitting materials, several strategies are generally applied to tune the energy gap in these materials. They involve the extension of the length of π-conjugation in unsaturated molecules^13^ and the introduction of metal centers into coordination compounds^8^, as well as the association of electron-donor and acceptor units in molecular materials^14,15^. Moreover, two-photon absorption (TPA) materials, through the absorption of two photons simultaneously, are capable of converting the excitation wavelengths from the visible to the NIR region. This property engenders an alternative approach for the construction of NIR absorbing materials. Two-photon excited NIR-emitting materials, with inherent multifunctionality, are ideal candidates for cell imaging^16^, photodynamic therapy^17,18^, microfabrication^19^, and optical data storage^20,21^. Consequently, the exploration of effective design strategies, along with the development of new two-photon excited NIR emissive materials, constitutes an attractive objective in materials science.

Recently, organic co-crystals, wherein donor and acceptor molecules assemble into highly ordered superstructures, based on intermolecular noncovalent bonding interactions, have emerged as a category of supramolecular materials with the following advantages^22–24^. (i) The preparation methods of co-crystals are similar to those of traditional single-component crystallization, which are feasible and easy to perform^25^. (ii) The properties of the co-crystals are related to their molecular packing and morphology, both of which can be modulated through co-crystal engineering without the requirement for further chemical synthesis^26^. (iii) Co-crystals not only maintain the properties of the individual components, but also, on some occasions, display unusual properties^27–29^. For instance, a co-crystal based on tetrathiafulvalene (TTF) and tetracyanoquinodimethane (TCNQ) exhibits metallic electrical conductivity, in sharp contrast with the semiconductor characteristics of their individual crystals^30^. Although low molecular weight organic ferroelectrics are few and far between, charge transfer co-crystals, with lock-arm supramolecular ordering (LASO) motifs, exhibit^31^ ferroelectric behavior at room temperature. Inspired by these advances, extensive investigations of organic co-crystals have been pursued by scientists in different areas of materials science^32,33^. To date, abundant properties, such as ambipolar charge transport^34^, optical waveguide modulation^35^, piezochromic behavior^36,37^, thermal-mechanical response^38^, photothermal conversion^39^, and room-temperature phosphorescence^40^, have been achieved by simple co-crystallization. The engineering of NIR emissive co-crystals that exhibit TPA, however, remains as a challenge.

**Fig. 1:**
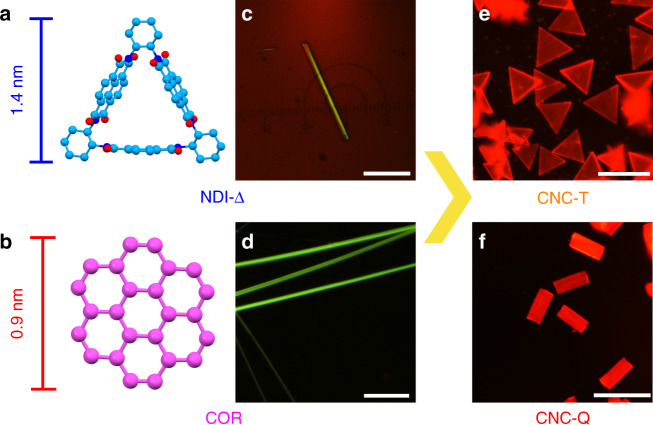
Crystal engineering of NDI-Δ, COR, CNC-T, and CNC-Q. **a** Solid-state structure of **NDI-Δ**. C light blue, N dark blue, O red. **b** Solid-state structure of **COR**. C pink. **c** Fluorescence microscopy image of an **NDI-Δ** crystal illustrating that it exhibits a 1D morphology and emits green fluorescence. Scale bar: 20 μm. **d** Fluorescence microscopy image of **COR** crystals, which display a 1D morphology and green fluorescence. Scale bar: 40 μm. **e** Fluorescence microscopy image of **CNC-T**, which exhibits a unique triangular morphology and red fluorescence. Scale bar: 50 μm. **f** Fluorescence microscopy image of **CNC-Q**, which displays a quadrangular morphology and red fluorescence. Scale bar: 50 μm.

Additionally, the organic naphthalenediimide-based triangle (Fig. 1a and Supplementary Figs. 2–7), namely **NDI-Δ**^41^, consisting of three electron-deficient NDI units, linked by *trans*-1,2-cyclohexano bridges, has been applied widely in supramolecular chemistry. On account of its well-defined inner cavity, **NDI-Δ** is capable^42^ of undergoing selective assembly into either helical or non-helical nanotubes upon the encapsulation of halogenated solvent molecules, e.g., XCH_2_CH_2_X where X=Cl, Br, I. The **NDI-Δ**, which is electron-deficient, is capable of encapsulating^41^ linear I_3_^−^ anions, resulting in the formation of single-handed helices in the solid state. Also, the electron-poor **NDI-Δ** and electron-rich TTF can, as a result of [π···π] interactions, co-assemble into two-dimensional (2D) crystalline superstructures^43^. On the other hand, **NDI-Δ**-based rechargeable lithium-ion batteries exhibit^44^ ultrahigh rate capability, as a result of the through-space electron sharing between NDI units in **NDI-Δ** molecules. The optical properties of **NDI-Δ** and its supramolecular assemblies, however, have hardly been investigated.

Herein, we examine a two-photon excited NIR emissive supramolecular material based on the co-crystallization of **NDI-Δ** and coronene (**COR**, Fig. 1b). Owing to the good match of their molecular sizes, the electron acceptor **NDI-Δ** and the electron donor **COR** can form two **COR**-**NDI-Δ** co-crystals (**CNC**s) with different morphologies—triangular and quadrangular in shape—which have been denoted by **CNC-T** and **CNC-Q**, respectively. The selective co-crystallization can be controlled by varying both the nature of solvents and the donor–acceptor stoichiometries. Analyses of the solid-state superstructures reveal that **CNC-Q** exhibits a more efficient donor–acceptor [π···π] overlap compared with that of **CNC-T**, which engenders a stronger charge-transfer interaction in **CNC-Q**. Consequently, **CNC-Q** has a narrower bandgap and emits deep-red and NIR fluorescence centered on 668 nm, while **CNC-T** displays deep-red fluorescence with a peak at 638 nm. The TPA properties of **NDI-Δ** and **COR** are retained in the co-crystals. Benefiting from intermolecular charge-transfer interaction, **CNC-T** and **CNC-Q** possess higher TPA cross-sections and longer two-photon excitation wavelengths that extend up into the NIR-II region when compared to their individual components. Overall, **CNC-T** and **CNC-Q**, not only constitute two rare co-crystals that exhibits TPA, as well as deep-red and NIR emission, but also serve as a powerful tool for uncovering the superstructure-property relationships. This investigation provides insights into tuning the optical properties in supramolecular systems and sheds light on the possibility of employing co-crystallization as a strategy for the production of multifunctional materials.

## Results

### Growth of NDI-Δ crystals and NDI-Δ-based co-crystals

**NDI-Δ** molecules are known^42^ to adopt different packing arrangements in different solvents, indicating that their selection wields a significant influence on the crystallization of **NDI-Δ** molecules. In order to slow down the rate of crystal growth and obtain high-quality crystals of **NDI-Δ**, PhCl was selected as the solvent because of its high boiling point. Upon slow evaporation of PhCl, colorless one-dimensional (1D) needle **NDI-Δ** crystals were obtained (Supplementary Fig. 19). Single-crystal X-ray diffraction (XRD) analysis of the crystals reveals (Supplementary Fig. 11) that the **NDI-Δ** molecules stack in 1D columns, wherein adjacent molecules interact by means of [C−H···O] hydrogen bonding between aromatic hydrogen and carbonyl oxygen atoms with distances ranging from 2.20 to 2.71 Å. Some PhCl molecules are included in the **NDI-Δ** columns, stabilized by a [Cl···π] interaction with a distance of 3.37 Å and [C−H···π] interactions covering distances of 2.66–2.90 Å. The powder XRD pattern (Supplementary Fig. 18a) of the **NDI-Δ** crystals is consistent with the simulated pattern based on the single-crystal XRD data, indicating the high quality of the **NDI-Δ** crystals.

**COR**, an aromatic electron donor with good optoelectrical properties (Supplementary Fig. 21)^45,46^, possesses a strong ability^38^ to interact with electron acceptors. Owing to the larger size of **COR** compared to that of the **NDI-Δ** cavity, **COR** is only capable of interacting with the outer surface of **NDI-Δ** by means of [π···π] interactions. This mode of packing will increase the [π···π] overlap throughout the entire superstructure, enriching the potential optoelectrical properties of the co-crystals. Hence **COR** is employed to enter into co-crystallization with electron-accepting **NDI-Δ** molecules. High-quality crystals of **COR** could be grown (Supplementary Fig. 18b) by slow evaporation of its CH_2_Cl_2_ solution. These crystals also exhibit (Supplementary Fig. 19) a 1D morphology.

The electron acceptor **NDI-Δ** and the **COR** donor, as a result of intermolecular charge transfer interactions, form (Supplementary Fig. 20) selectively two **COR**-**NDI-Δ** co-crystals (**CNC**s). One of them exhibits a unique triangular morphology, and the other possesses a quadrangular shape (Supplementary Fig. 19). Based on their morphologies, the two **CNC**s were named **CNC-T** and **CNC-Q**, respectively. The **CNC**s were found to be sensitive to the crystallization solvents and the respective donor–acceptor stoichiometries used in their preparation. For example, with a donor–acceptor molar ratio of 1:2, **CNC-T** can be obtained (Supplementary Fig. 18c) selectively by slow vapor diffusion of MeOH into a THF solution. Alternatively, with a donor–acceptor molar ratio of 1:1, **CNC-Q** can be obtained (Supplementary Fig. 18d) selectively by slow vapor diffusion of Et_2_O into a CH_2_Cl_2_ solution.

The fluorescence microscopy images reveal (Fig. 1c–f) that both **CNC-T** and **CNC-Q** emit bright red fluorescence, which is significantly different from the green fluorescence of individual **NDI-Δ** and **COR** crystals, indicating the influence of co-crystallization upon the optical behavior. With identical supramolecular co-constitutions but different donor–acceptor stoichiometries, **CNC-T** and **CNC-Q** serve as ideal models to unravel the superstructure-property relationships^47^ in these co-crystals. Meanwhile, reports relating to the influence of donor–acceptor stoichiometry on the luminescence behavior of co-crystals are rare^48,49^. These facts motivated us to gain in-depth understanding to the superstructures and optical properties of **CNC-T** and **CNC-Q**.

### Solid-state superstructures and morphologies of co-crystals

**Fig. 2:**
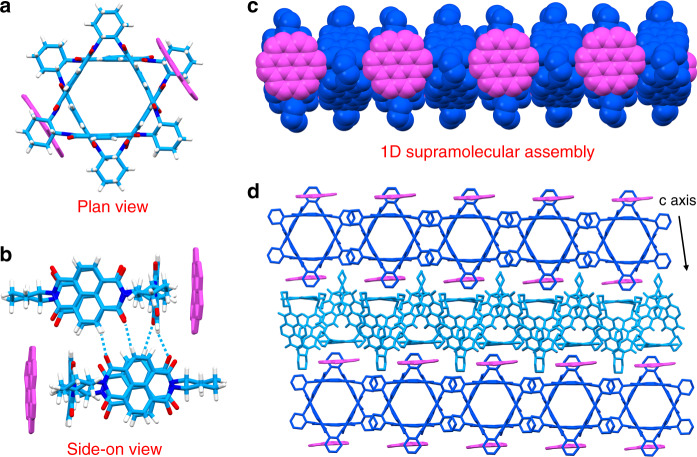
Solid-state superstructure of **CNC-T**. **a** Plan view of a capped-sticks representation demonstrating the face-to-face packing between **COR** and **NDI-Δ**. **b** Side-on view of a capped-sticks representation indicating that two adjacent **NDI-Δ** molecules are linked by [C–H···O] hydrogen bonding. **c** 1D Columnar superstructure composed of inner **NDI-Δ** nanotube and outer **COR** molecules. **d** Solid-state superstructure of **CNC-T** illustrating the layer-by-layer stacking along the *c* axis.

Single-crystal XRD analysis of **CNC-T**, in which the molar ratio between the donor and acceptor is 1:2, reveals that it belongs (Supplementary Table 3) to the monoclinic *C*2 space group. **NDI-Δ** and **COR** adopt (Fig. 2a) a face-to-face stacking mode based on [π···π] and [C−H···π] interactions. The plane-to-plane distances of the donor–acceptor [π···π] interactions between the **COR** and one of the NDI units range from 3.24 to 3.38 Å. The [C−H···π] interactions refer to (Supplementary Fig. 13) the cyclohexano hydrogen atoms in **NDI-Δ** and the plane of **COR** with distances ranging from 2.60 to 2.88 Å. The donor–acceptor pairs of **COR** and **NDI-Δ** stack in a 1D columnar superstructure (Fig. 2c), through [C−H···O] hydrogen bonding between aromatic hydrogen and carbonyl oxygen atoms in two adjacent **NDI-Δ** molecules (Fig. 2b) with distances ranging from 2.31 to 2.59 Å. These 1D columns pack into a 2D layer in the *a*−*b* plane (Supplementary Fig. 14c) by means of [C−H···π] interactions (Supplementary Fig. 13) between cyclohexano hydrogen atoms and the NDI plane in neighboring **NDI-Δ** molecules with distances in the range of 2.69–2.90 Å. The 2D layers are tightly packed (Fig. 2d) along the *c* axis and form a well-ordered three-dimensional (3D) array.

**CNC-T** displays a triangular morphology, an observation which is not common in organic crystals. In order to gain more insight into the formation of this geometry, powder XRD analysis and morphology simulation were carried out on **CNC-T**. The powder XRD pattern of **CNC-T**, grown on a glass substrate, displays (Supplementary Fig. 18c) strong (001) and (002) diffraction peaks, indicating^35^ that the triangular growth face of the co-crystal is parallel to the (00l) lattice plane. This experimental data is confirmed by the simulated morphology of the co-crystals. **CNC-T** possesses (Supplementary Fig. 14) an isosceles triangular geometry, wherein the (001) and (00–1) growth faces account for (Supplementary Table 1) the largest facet area percentage of 42.5% as a result of their lowest attachment energy (*E*_att_). The vertex angle of a simulated isosceles triangle is 62°, an angle which is consistent with that (62°) between the (110) and (1–10) lattice planes observed (Supplementary Fig. 15) in the single-crystal XRD data. Benefiting from the high symmetry in the *C*2 space group, the (110) and (1–10) growth faces display identical *E*_att_ and the exposed facet area percentage in the simulated morphology, so that **CNC-T** adopts an isosceles triangular geometry.

**Fig. 3:**
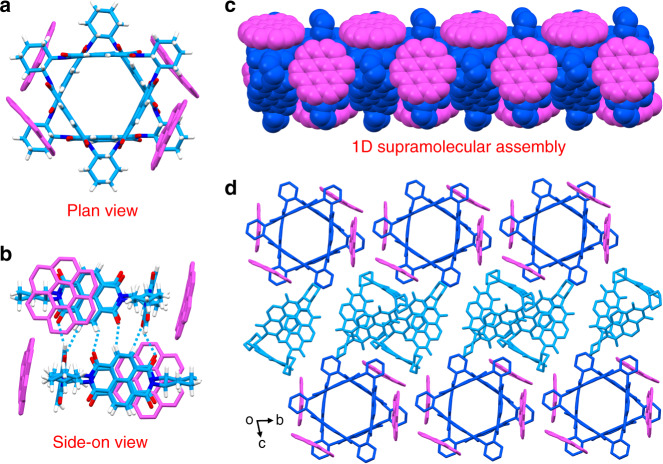
Solid-state superstructure of **CNC-Q**. **a** Plan view of a capped-sticks representation illustrating that **NDI-Δ** and **COR** molecules adopt a face-to-face packing motif. **b** Side-on view demonstrating [C–H···O] hydrogen bonding involving two neighbor **NDI-Δ** molecules. **c** 1D Columnar superstructure composed of inner **NDI-Δ** nanotube and outer **COR** molecules. **d** Solid-state superstructure of **CNC-Q** revealing the layer-by-layer stacking along the *c* axis.

In contrast, **CNC-Q**, with a 1:1 donor–acceptor stoichiometry, crystallizes (Supplementary Table 3) in a triclinic *P*1 space group. With the change in donor–acceptor ratio, **NDI-Δ** molecules are surrounded (Fig. 3a) by one more equivalent of **COR** molecules in **CNC-Q** than that observed in **CNC-T**. This stoichiometry variation, along with the through-space electron sharing between NDI units in the **NDI-Δ**^41^ (Supplementary Fig. 8), results in **CNC-Q** exhibiting a more efficient donor–acceptor overlap. In the solid-state superstructure of **CNC-Q**, **NDI-Δ** and **COR** molecules also exhibit (Supplementary Fig. 16a) face-to-face stacking stabilized by [π···π] and [C−H···π] interactions with distances of 3.29−3.39 and 2.74−2.90 Å, respectively. The 1D columnar supramolecular assembly, composed of an inner **NDI-Δ** nanotube and a layer of outer **COR** molecules, is also formed (Fig. 3b, c) along the *a* axis, supported by [C−H···O] hydrogen bonds with distances ranging from 2.40 to 2.60 Å. These 1D colunms stack (Fig. 3d) in 2D layers in the *a–b* plane (Supplementary Fig. 17d) interconnected by **COR** molecules through (Supplementary Fig. 16a) [π···π] and [C−H···π] interactions. Subsequently, these 2D layered nanostructures are held together by coaxial **NDI-Δ** dimers (Supplementary Fig. 16b), relying on [π···π] interactions, to form (Fig. 3d) well-ordered 3D arrays along the *c* axis.

Differing from the triangular morphology of **CNC-T**, **CNC-Q** exhibits a quadrangular geometry with an angle of 95°. The value is the same as that observed (Supplementary Fig. 17) from the (011) and (0−1−1) growth faces in the simulated morphology of **CNC-Q**. According to the theoretical calculations, the *E*_att_ of (001) and (00−1), (011) and (0−1−1), (100) and (−100) growth faces are (Supplementary Table 2) −64.3, −66.4, and −81.0 kcal/mol, respectively. These energies lead to the percentages of their exposed facet areas in the simulated morphology being 34.6, 33.6, and 26.8%, respectively, explaining why we observe the quadrangle-shaped **CNC-Q**.

In summary, comparing these two co-crystals, we can conclude that (i) **NDI-Δ** and **COR** prefer to adopt a face-to-face packing motif driven by charge-transfer interactions even in different solvents, (ii) **NDI-Δ** is prone to assemble into 1D supramolecular arrays supported by multiple [C−H···O] hydrogen bonds, and (iii) alteration in donor–acceptor stoichiometries results in dramatic differences in the solid-state superstructures and morphologies.

### Absorption and fluorescence spectra

**Fig. 4:**
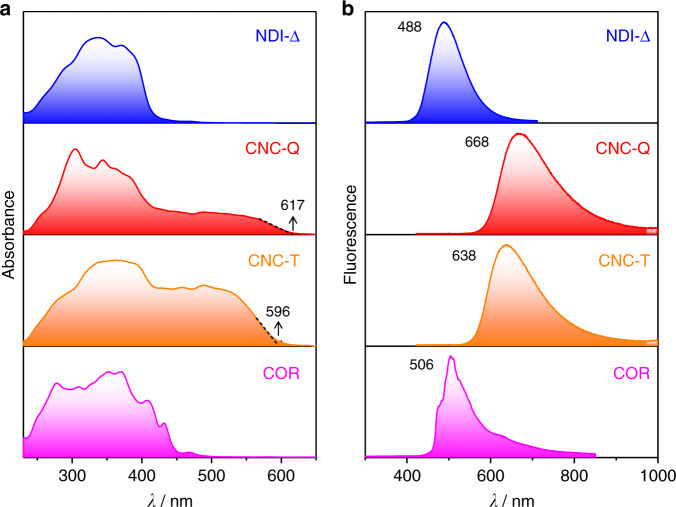
Solid-state spectroscopic characterization. Solid-state **a** UV-Vis absorption and **b** fluorescence spectra of **NDI-Δ**, **CNC-Q**, **CNC-T** and **COR**. The spectra of **CNC-T** and **CNC-Q** are significantly red-shifted compared to those of **NDI-Δ** and **COR** crystals.

In order to elucidate the photophyscial properties of **CNC-T** and **CNC-Q**, solid-state UV-Vis absorption and fluorescence spectra were recorded. **NDI-Δ** crystals show (Fig. 4a) strong absorption in the region of 240–420 nm, and **COR** crystals absorb primarily at 240–450 nm. Distinctive absorption spectra were observed after co-crystallization. **CNC-T** exhibits a broad absorption ranging from 240 to 596 nm, which is significantly red-shifted in comparison with the individual **NDI-Δ** and **COR** crystals, on account of the intermolecular charge-transfer interaction in the co-crystal. By contrast, the absorption band of **CNC-Q** extends up to 617 nm, which is 21 nm red-shifted compared to that of **CNC-T**. This difference may arise from a more efficient donor–acceptor [π···π] overlap in the solid-state superstructure of **CNC-Q** than in that of **CNC-T**.

Fluorescence spectra reveal (Fig. 4b) that the two co-crystals display a red-shifted emission compared to that of the individual molecular crystals, an observation which is in agreement with the fluorescence microscopy images (Fig. 1). **NDI-Δ** and **COR** crystals show green fluorescence with emission peaks centered on 488 and 506 nm, respectively. Their corresponding solid-state photoluminescence quantum yields (PLQYs, *Ф*_*F*_) are 1.4 and 3.2%. In comparison with their solution-state fluorescence spectra (Supplementary Fig. 21), the solid-state fluorescence spectra of **NDI-Δ** and **COR** crystals are red-shifted, on account of their particular packing modes in the solid state^50^. **NDI-Δ** molecules have a rigid triangular geometry, which prevents the formation of H-type aggregates, while **COR** molecules adopt (Supplementary Fig. 12) a slipped face-to-face packing mode with J-type aggregation in their crystalline state. **CNC-T** exhibits (Fig. 4b) deep-red fluorescence^51^ with a peak at 638 nm, while **CNC-Q** displays (Fig. 4b) deep-red and NIR fluorescence with a maximum emission wavelength at 668 nm, which is red-shifted by 162 nm compared to that of the **COR** crystals. The solid-state PLQYs of **CNC-T** and **CNC-Q** are 0.9 and 2.2%, respectively, values which are comparable to those of the individual component crystals. These observations underline the viability of the co-crystallization strategy in producing solid-state NIR functional materials^11,52^. Time-resolved fluorescence measurements reveal that both **CNC-T** and **CNC-Q** exhibit (Supplementary Fig. 22) a double-exponential fluorescence decay curve with an average lifetime of 11.4 and 9.7 ns, respectively. The radiative rate constant (*k*_r_) and non-radiative rate constant (*k*_nr_) of **CNC-T** are (Supplementary Table 4) 7.9 × 10^5^ and 8.7 × 10^7^ s^−1^, respectively, while those for **CNC-Q** are 2.3 × 10^6^ and 1.0 × 10^8^ s^−1^. The larger *k*_r_ and *k*_nr_ values of **CNC-Q** are consistent with its faster fluorescence decay lifetime when compared with that of **CNC-T**. These observations indicate that the photophysical properties of co-crystals are sensitive to their solid-state superstructures. On account of the different donor–acceptor stoichiometry and the through-space electron sharing between NDI units in the **NDI-Δ**^41^ (Supplementary Fig. 8), **CNC-Q** exhibits more efficient donor–acceptor overlap and stronger intermolecular charge-transfer interactions compared with **CNC-T**. Consequently, **CNC-Q** displays the more red-shifted absorption and emission spectra.

### Frontier molecular orbitals calculations

**Fig. 5:**
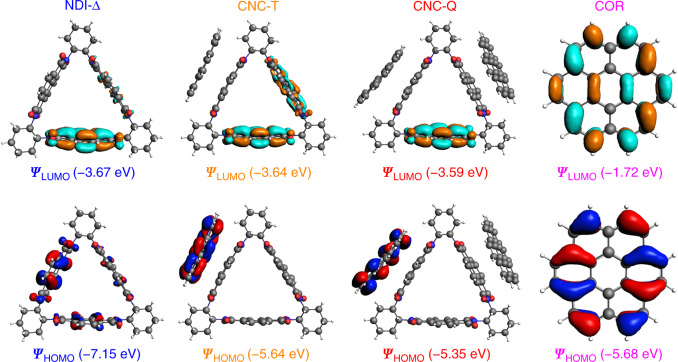
DFT Calculations. Frontier molecular orbitals of **NDI-Δ**, **CNC-T**, **CNC-Q** and **COR** along with their corresponding energy gaps, which are 3.47, 2.00, 1.76 and 3.97 eV, respectively.

In order to attain a deeper understanding of the electronic structures of the co-crystals, density functional theory (DFT) calculations were conducted (Supplementary Figs. 31–34) with the B3LYP functional and an all-electron triple zeta basis set. The frontier molecular orbital diagrams demonstrate (Fig. 5) that the highest occupied molecular orbitals (HOMOs) of **CNC-T** and **CNC-Q** are concentrated on the electron-donating **COR** molecule with energies of −5.64 and −5.35 eV, respectively. These values are close to that of **COR** (−5.68 eV). The lowest unoccupied molecular orbitals (LUMOs) of **CNC-T** and **CNC-Q** are localized on the electron-accepting **NDI-Δ** molecule with energies of −3.64 and −3.59 eV, respectively, resembling that of **NDI-Δ** (−3.67 eV). These observations indicate that the HOMO of co-crystal is primarily derived from the HOMO of the donor, while the LUMO of co-crystal is chiefly dependent on that of the acceptor. The calculated HOMO-LUMO energy gaps (Δ*E*) of **CNC-T** and **CNC-Q** are 2.00 and 1.76 eV, respectively. This tendency is in agreement with the results obtained (Δ*E* = 2.09 and 2.01 eV for **CNC-T** and **CNC-Q**, respectively) from UV-Vis absorption spectra. The reason for the narrower bandgap of **CNC-Q** arises from the change of donor–acceptor ratio from 1:2 in **CNC-T** to 1:1 in **CNC-Q**. The results demonstrate that the stoichiometric variation leads to the changes in supramolecular arrangements, hence tuning the electronic structures and photophysical properties of the co-crystals.

### Two-photon absorption

**Fig. 6:**
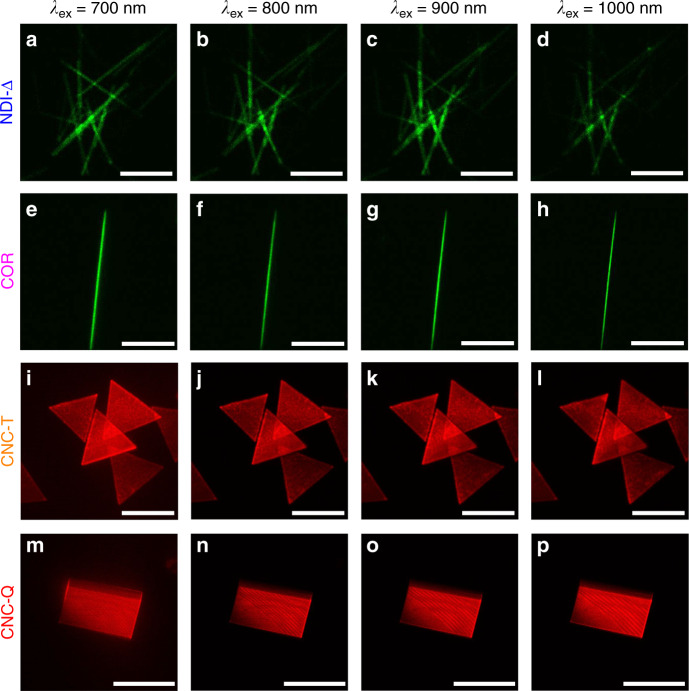
Photon upconversion characteristics. Two-photon microscopy images of **a**−**d NDI-Δ**, **e−h COR**, **i**−**l** **CNC-T** and **m**−**p CNC-Q**. **a**−**d** The excitation laser powers for **NDI-Δ** crystals were 7.0, 30.4, 54.0, and 54.0 mW, respectively. Scale bar: 20 μm. **e**−**h** The excitation laser powers for **COR** crystal were 6.7, 37.1, 72.0, and 54.0 mW, respectively. Scale bar: 100 μm. **i**−**l** The excitation laser powers for **CNC-T** were 6.3, 8.1, 3.5, and 2.7 mW, respectively. Scale bar: 40 μm. **m**−**p** The excitation laser powers for **CNC-Q** were 3.0, 3.8, 1.6, and 1.1 mW, respectively. Scale bar: 100 μm.

**Fig. 7:**
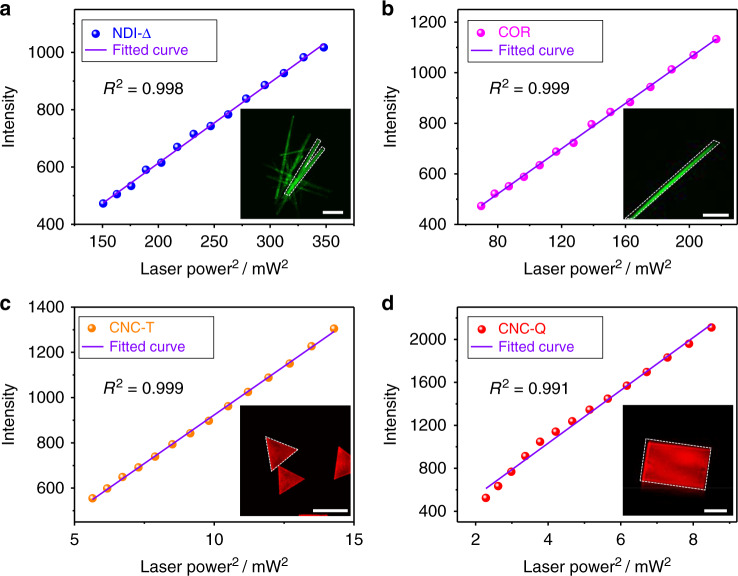
Excitation power dependence of upconversion fluorescence intensity. The linear dependence between the upconversion fluorescence intensity and the square of the excitation laser power for **a NDI-Δ** excited at 740 nm, **b COR** excited at 740 nm, **c CNC-T** excited at 1000 nm and **d CNC-Q** excited at 1000 nm. The upconversion fluorescence intensity of each material was collected from the marked region in the inset two-photon microscopy image. Scale bars: 20 μm in (**a**, **b**, **d**), 50 μm in (**c**).

TPA is a third-order nonlinear optical process, which occurs upon exposure of materials to high-intensity lasers. Upon excitation by a 700 nm laser, **NDI-Δ** crystals were observed (Fig. 6a) by a two-photon microscope. Their emission was collected in the green detection channel (detection region: 470–550 nm), corresponding closely to their fluorescence emission (488 nm). Similar fluorescence emission by **NDI-Δ** crystals can also be detected (Fig. 6b–d) in the green channel of the two-photon microscope when excited at 800, 900, and 1000 nm. These observations indicate that **NDI-Δ** crystals are capable of emitting light at a shorter wavelength upon excitation at a longer wavelength, a process which corresponds to photon upconversion. When the excitation wavelength is fixed at 740 nm, the fluorescence intensity of **NDI-Δ** crystals is proportional to the square of the incident laser power (Fig. 7a and Supplementary Fig. 23), implying that the upconversion emission of **NDI-Δ** crystals originates from a TPA process^53^. **COR** crystals display a similar TPA process (Fig. 6e–h) to that of **NDI-Δ** crystals. Upon excitation at 700, 800, 900, and 1000 nm, the upconversion emission by **COR** crystals, which agrees with their fluorescence emission (506 nm), is also collected in the green detection channel. At a fixed excitation wavelength of 740 nm, the fluorescence intensity of **COR** crystals increases (Fig. 7b and Supplementary Fig. 24) linearly with the square of laser power, indicating an active TPA process.

Similarly, the two co-crystals also exhibit nonlinear optical responses. At laser irradiation from 700 to 1000 nm, the upconversion emission of **CNC-T** is collected (Fig. 6i–l) in the red detection channel (detection region: 550–650 nm), which corresponds closely to the fluorescence emission (638 nm) of **CNC-T**. At a fixed excitation wavelength of 1000 nm, the fluorescence intensity of **CNC-T** displays (Fig. 7c and Supplementary Fig. 25) a linear dependence on the square of the laser power. The TPA of **CNC-Q** is also detected (Fig. 6m–p) by two-photon imaging in the red channel, in accordance with its fluorescence emission band in the region of 580–950 nm. The corresponding excitation power dependence of fluorescence intensity is illustrated in Fig. 7d and Supplementary Fig. 26. In addition, the TPA of the co-crystals is further supported by time-dependent density-functional theory (TDDFT) calculations (Supplementary Fig. 35 and Table 6). In the calculated one-photon absorption spectra, the lowest-energy excited state of **CNC-T** corresponds to the wavelength of 677 nm with a negligible oscillator strength of 0.009 a.u.. **CNC-Q** exhibits three lowest-energy excitations at the wavelength of 725, 770, and 827 nm, with quite small oscillator strengths of 0.027, 0.001, and 0.020 a.u., respectively. Consequently, the emission of the co-crystals at excitation wavelengths of ~1000 nm stems mainly from the TPA rather than the one-photon absorption of their lowest-energy excited states.

**Fig. 8:**
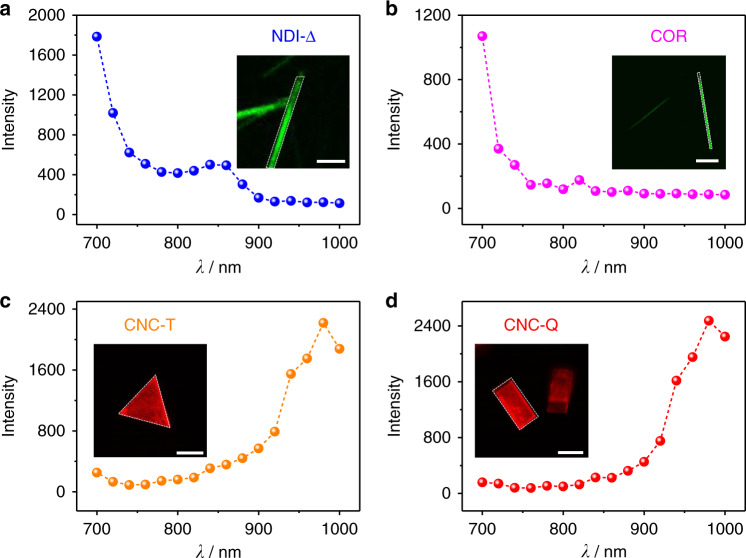
Excitation wavelength dependence of upconversion fluorescence intensity. Two-photon excited spectra of **a NDI-Δ**, **b COR**, **c CNC-T** and **d CNC-Q**. The laser powers corresponding to the spectra were set to be 13.3, 2.7, 2.7, and 2.7 mW, respectively. The upconversion fluorescence intensity of each material was collected from the marked region in the inset two-photon microscopy image. Scale bars: 10 μm in (**a**), 100 μm in (**b**), 20 μm in (**c**, **d**).

Taking into consideration that the TPA is governed by different quantum-mechanical rules, in comparison with one-photon absorption, the TPA spectra can display distinct line shapes compared to their one-photon spectra^54^. The two-photon excitation spectra of these crystals were recorded to explore the excitation wavelength dependence of their upconversion emission intensity (Supplementary Figs. 27–30). For this purpose, an in-situ two-photon imaging experiment was conducted at a constant laser power, while the laser excitation wavelengths were varied in the range of 700–1000 nm by intervals of 20 nm. The upconversion emission intensity was collected from the same sample region and then plotted as the function of the excitation wavelength. The resulting two-photon excitation spectra (Fig. 8) reveal that **NDI-Δ** and **COR** crystals display the strongest upconversion fluorescence at 700 nm. In sharp contrast, the two-photon excitation spectra of **CNC-T** and **CNC-Q** exhibit a similar shape with peaks centered on 980 nm. These profiles indicate that co-crystallization leads to a red-shifted TPA spectrum compared to the individual molecular crystals. Remarkably, **CNC-Q** serves as a co-crystal that exhibits two-photon response and NIR emission simultaneously, implying that the co-crystallization strategy serves as a convenient and efficient design approach towards multifunctional optical materials.

### Two-photon excitation spectra simulations

**Fig. 9:**
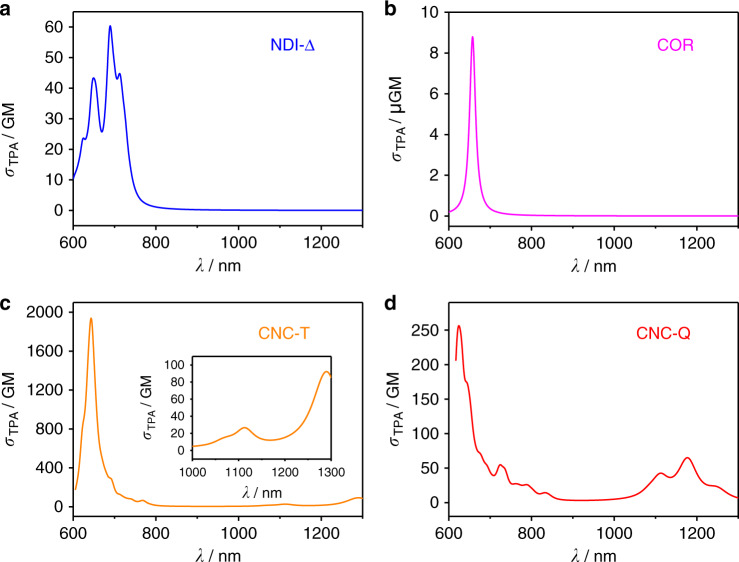
Second linear response TDDFT calculations. The simulated two-photon excited spectra of **a NDI-Δ**, **b COR**, **c CNC-T** and **d CNC-Q**. The inset in **c** displays a magnified simulated two-photon excited spectrum of **CNC-T** in the region of 1000–1300 nm.

Second linear response TDDFT calculations were performed using an in-house code to gain a theoretical understanding of the TPA spectrum^55,56^. The calculated TPA spectra (Fig. 9) of all four materials are consistent with the experimental results (Fig. 8). The **NDI-Δ** crystals display a broad TPA spectrum in the region of 600–800 nm, and exhibit a maximum TPA cross-section (σ_TPA_) of 60 GM at 690 nm. The **COR** crystals display a sharp two-photon excitation peak at 658 nm with a weak σ_TPA_ of 8.8 μGM. The maximum σ_TPA_ of **CNC-T** and **CNC-Q** are 1939 GM at 643 nm and 256 GM at 624 nm, respectively. These values are 32 and 4.3 times larger than that for **NDI-Δ** crystals. **CNC-T** possesses higher σ_TPA_ than that for **CNC-Q**, a difference which may arise from the fourth-power dependency of σ_TPA_ on the transition dipoles and the average values of transition dipoles for **CNC-T** are 48% higher than those for **CNC-Q**. Moreover, in the calculated TPA spectra of the co-crystals, a new absorption band was identified in the range from 1000 to 1300 nm in the NIR-II region^57,58^, with a maximum σ_TPA_ of 92 GM at 1290 nm and 65 GM at 1177 nm for **CNC-T** and **CNC-Q**, respectively. The possible reasons for the increased TPA cross-sections and red-shifted TPA spectra of co-crystals include the following. (i) The donor and acceptor molecules adopt a face-to-face packing motif, facilitating the [π···π] overlap throughout the entire superstructure. (ii) The intermolecular charge-transfer interaction induces the π-electron delocalization from the donor to the acceptor molecules, contributing to the supramolecular electronic polarization in the co-crystals^53,59^. These results imply that the design of charge transfer co-crystals emerges as an alternative approach for the synthesis of TPA materials with enhanced two-photon response. In the rational design of two-photon excited NIR emissive co-crystals, two fundamental points can be taken into consideration—namely, selecting TPA-active precursors in order to induce nonlinear optical properties, and introducing intermolecular charge-transfer interactions in order to achieve NIR emission and enhanced two-photon responses.

## Discussion

We have applied a supramolecular approach to the design of two-photon excited deep-red and NIR emissive materials based on the co-crystallization of **NDI-Δ** and **COR**. Two charge transfer co-crystals, **CNC-T** and **CNC-Q**, have been obtained by controlling the nature of the solvents and the donor–acceptor stoichiometries. These co-crystals provide an example of how to gain better insights, through experiments supported by theory, into the relationship between the superstructures of materials and their properties. By altering the donor–acceptor ratio from 1:2 (**CNC-T)** to 1:1 (**CNC-Q)**, the intermolecular charge-transfer interactions become stronger and the energy bandgap becomes narrower in **CNC-Q**. Hence **CNC-Q** exhibits deep-red and NIR emission centered on 668 nm, while **CNC-T** emits deep-red fluorescence with peak at 638 nm. The TPA properties of the co-crystals are enhanced compared to those of the individual molecular crystals because of the intermolecular charge-transfer interactions. The **CNC-T** and **CNC-Q** co-crystals, not only exhibit red-shifted TPA spectra in the NIR-II region, but also possess larger TPA cross-sections. Notably, the **CNC-Q** co-crystal emerges as an innovative two-photon excited NIR-emissive supramolecular material. The co-crystallization strategy presented here possesses the distinct advantage that comes with noncovalent synthesis, providing as it does a facile modular approach to advanced smart materials with multiphoton absorption and tunable emission properties.

## Methods

### Materials

All chemicals and solvents were purchased from commercial companies and used directly without further purification. ***(RRRRRR)*-NDI-Δ**—namely **NDI-Δ** employed in this research—was synthesized and purified following methods^41^ as shown in Supplementary Fig. 1. **Coronene** (C_24_H_12_, 97%), defined as **COR**, was purchased from Sigma-Aldrich. Nuclear magnetic resonance (NMR) spectra were recorded on a Agilent 500 MHz spectrometer with a working frequency of 500 MHz in the case of ^1^H NMR spectroscopy. Chemical shifts are reported in ppm relative to the signals corresponding to the residual non-deuterated solvents (CDCl_3_: δ 7.26 ppm).

### Preparation of co-crystal CNC-T

**NDI-Δ** (20.8 mg, 20 μmol) was first of all mixed with **COR** (3.0 mg, 10 μmol) in THF (20 mL) and dissolved by ultrasonic treatment (Supplementary Fig. 9). The solution was filtered with a 0.22-μm syringe filter to remove insoluble impurities. With slow vapor diffusion of MeOH into the filtered solution, high quality red triangle-shaped co-crystals formed after one week.

### Preparation of co-crystal CNC-Q

**NDI-Δ** (20.8 mg, 20 μmol) was first of all mixed with **COR** (6.0 mg, 20 μmol) in CH_2_Cl_2_ (20 mL) and dissolved by ultrasonic treatment (Supplementary Fig. 10). The solution was filtered with a 0.22-μm syringe filter to remove the insoluble impurities. With slow vapor diffusion of Et_2_O into the filtered solution, high quality red quadrangle-shaped co-crystals formed after one week.

### Optical and fluorescence microscopies

Optical and fluorescence microscopy images were obtained at room temperature with a Nikon LV150, which was equipped with a high-intensity white light LED source and a 5 mega pixel color camera for image acquisition. Optical microscopy pictures were captured in the bright field mode. Fluorescence microscopy images were captured in the fluorescence mode.

### Powder X-ray diffraction analysis

Powder X-ray diffraction (PXRD) was carried out on a STOE STADI MP powder diffractometer, which was equipped with an asymmetric curved Germanium monochromator (Cu *K*_*α1*_ radiation, λ = 1.54056 Å) and a one-dimensional silicon strip detector (MYTHEN2 1K from DECTRIS). The line focused Cu X-ray tube was operated at 40 kV and 40 mA. Samples of micro/nano-crystals were grown on glass substrates. **NDI-Δ** crystals were prepared by slow evaporation from a PhCl solution. **COR** crystals were prepared by slow evaporation from a CH_2_Cl_2_ solution. **CNC-T** was prepared by slow vapor diffusion of MeOH into a THF solution with a 1:2 donor–acceptor molar ratio. **CNC-Q** was prepared by slow vapor diffusion of Et_2_O into a CH_2_Cl_2_ solution with a 1:1 donor–acceptor molar ratio. Paratone oil was used to protect the crystals. Experiments were carried out in reflection geometry mode at room temperature. The simulated powder XRD patterns with Cu *K*_*α1*_ radiation were carried out by Mercury software 4.3.1.

### Photophysical characterization

Solution UV-Vis absorption spectra were measured on a UV-3600 Shimadzu spectrophotometer at room temperature. Solution fluorescence spectra were recorded on a HORIBA FluoroMax-4 spectrometer at room temperature. Solid UV-Vis absorption spectra were measured in reflection mode at room temperature on a Shimadzu 3600 UV-visible-NIR spectrophotometer equipped with an integrating sphere. Solid fluorescence spectra were performed with a HORIBA Fluorolog-3 spectrometer. Fluorescence lifetime measurements and solid-state absolute photoluminescence quantum yields were obtained using a HORIBA FluoroMax-4 spectrometer equipped with an integrating sphere (HORIBA Quanta–φ).

### Raman spectroscopy

Raman spectra were carried out on a HORIBA LabRAM HR Evolution Confocal Raman instrument. The system was equipped with (i) multi-laser (473, 532, 633, and 785 nm), (ii) a confocal microscope for high spatial resolution, (iii) a Raman spectrometer detector for high spectral resolution and wide spectral range analysis, (iv) a high-precision DuoScan imaging instrument and (v) an electron multiplying CCD in order to enhance the measured S/N ratios.

### Cyclic voltammetry

Cyclic voltammetry (CV) was performed on a Gamry Multipurpose instrument (Reference 600) interfaced to a PC under N_2_ atmosphere at 298 K. The CV experiments were recorded with a glassy carbon working electrode (0.071 cm^2^, Cypress system). The electrode surface was polished routinely with alumina–water slurry on a felt surface before use. The counter electrode was a Pt coil, and the reference electrode was Ag/AgCl. CV Experiments were carried out in a 0.1 M solution of [Bu_4_N][PF_6_] electrolyte in CH_2_Cl_2_ at a 100 mV/s scan rate. The sample concentration was 0.5 mM.

### Two-photon microscopy

The upconversion emission and TPA-related measurements were performed on a Nikon A1R-MP^+^ Multiphoton Microscope with penetration depth up to 1.5 mm. The microscope was equipped with a tunable Chameleon Vision titanium sapphire laser from 700 to 1000 nm. In addition, the GaAsP detectors were applied for increased sensitivity. The resonant scanner was capable of scanning up to ~400 frames per second.

### Density functional theory computational methods

Structures for the single-molecule calculations were obtained from X-ray single crystal diffraction data. All electron density functional theory (DFT) single point calculations were performed at the B3LYP/TZP level in the Amsterdam Density Functional program (ADF, version 2018.104)^60–62^. The orbital surfaces were visualized with ADFView.

The TPA spectra were computed by second linear response TDDFT (SLR-TDDFT), an in-house code implemented within the NWChem suite^63^. This theory computes transition dipoles between different excited states with a computational cost similar to that of traditional linear-response (LR) TDDFT calculations. SLR-TDDFT Calculations can be performed by either “relaxing” the orbitals of the system, or by utilizing an unrelaxed scheme in which the excited-state transition dipoles are determined by a simple numerical differentiation procedure^55^. Given the size of the molecules, in this work the unrelaxed method was used on account of its computational efficiency. In principle, the unrelaxed method is equivalent to employing the auxiliary Casida wave functions^64^, but they can be reliably accurate^65^. For these calculations, the exchange-correlation functional LDA0 was used^66^. This functional—for optical properties—is as accurate as B3LYP, and it provides an additional speed-up for the calculations, which is critical in this case, given the relatively large number of atoms and excited states, especially for **CNC-Q**. The SLR-TDDFT requires as a first step a regular LR-TDDFT calculation under the Tamm-Dancoff approximation. Also, for computational convenience, the double-*ζ* basis set and effective-core potential SBKJC were applied. The Davidson convergence threshold is 10^−3^ for **COR** and **NDI-Δ**, and 10^−2^ for **CNC-T** and **CNC-Q**. This requirement guarantees the energies are accurate up to a fraction of a meV. In the LR-TDDFT calculation, we neglect virtual orbitals with energies above 12 and 8 eV for **CNC-T** and **CNC-Q**, respectively. These ranges are sufficient to cover the TPA spectra: the excited state with largest energy is below 4.14 eV. The SLR-TDDFT algorithm provides all the transition dipoles required to compute the TPA cross-section as a function of the photon wavelength. We assume random circularly polarized light interaction. The frequency-dependent TPA cross-section was computed using the formula for this type of light interaction^67,68^. We assume a Lorentzian line shape function, a broadening factor of 0.05 eV for virtual states, and 0.1 eV for the final states.

## Supplementary information

Supplementary Information

## Data Availability

All the data supporting the conclusions are included in this article and its Supplementary files, or are available from the authors upon reasonable request. The single-crystal diffraction data for **NDI-Δ**, **CNC-T** and **CNC-Q** have all been deposited in the Cambridge Crystallographic Data Centre (CCDC). The deposition numbers are CCDC 2004158–2004160, respectively.

## Code availability

The two-photon absorption post-processing code used in this article is available at https://gitlab.com/plusxmartin/insomnia.
