# Supplementary material for: Two-photon excited deep-red and near-infrared emissive organic co-crystals
Source: Nat Commun. 2020 Sep 15;11:4633. doi: 10.1038/s41467-020-18431-7 (PMC7493989; doi:10.1038/s41467-020-18431-7)
Supplement: Supplementary file 1 — Supplementary Information [file 41467_2020_18431_MOESM1_ESM.pdf]

Supplementary Information for

Two-photon excited deep-red and near-infrared emissive organic co-crystals

Yu Wang[1,4], Huang Wu[1,4], Penghao Li[1], Su Chen[1], Leighton O. Jones[1], Martín A. Mosquera[1], Long Zhang[1], Kang Cai[1], Hongliang Chen[1], Xiao-Yang Chen[1], Charlotte L. Stern[1], Michael R. Wasielewski[1], Mark A. Ratner[1], George C. Schatz[1] & J. Fraser Stoddart[1,2,3]*

[1]*Department of Chemistry, Northwestern University, 2145 Sheridan Road, Evanston, Illinois 60208, United States*

[2]*School of Chemistry, University of New South Wales, Sydney, NSW 2052, Australia*

[3]*Institute for Molecular Design and Synthesis, Tianjin University, 92 Weijin Road, Nankai District, Tianjin 300072, P.R. China*

[4]*These authors contributed equally*

*E-mail: stoddart@northwestern.edu

Table of Contents

Supplementary Note 1. Supplementary Synthetic Procedures S2

Supplementary Note 2. Crystallographic Characterization and Calculations S6

Supplementary Note 3. Powder X-Ray Diffraction Analysis S14

Supplementary Note 4. Photophysical Characterization S15

Supplementary Note 5. Two-Photon Absorption Analysis..................................... S17

Supplementary Note 6. Density Functional Theory Calculations........................... S23

Supplementary References... S28

Supplementary Note 1. Supplementary Synthetic Procedures

(1) Synthesis of the NDI-Δ

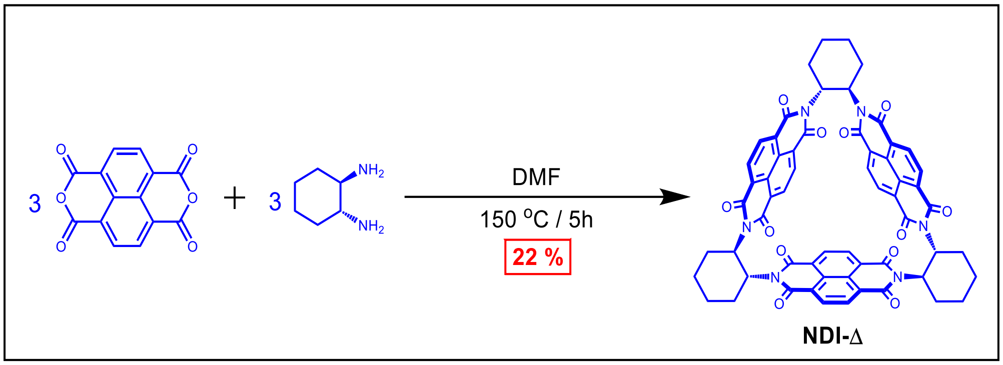

Supplementary Figure 1. Synthesis of **NDI-Δ**

NDI-Δ. An equimolar amount of *(RR)*-*trans*-1,2-cyclohexanediamine (1.7 g, 15 mmol) in anhydrous DMF (10 mL) was added quickly to a solution of naphthalenetetracarboxylic dianhydride (4.0 g, 15 mmol) in anhydrous DMF (200 ml) with vigorous stirring at 150 °C. After heating under reflux for 5h, the resulting reaction mixture was cooled to room temperature, and the DMF was evaporated under vacuum. The deep red residue was dissolved in CH_2Cl_2 and purified by flash column chromatography on silica gel (CH_2Cl_2 / Me_2CO, 0–10% Me_2CO), followed by precipitation of the product with MeOH to afford pure **NDI-Δ** in a yield of 22%. ^{1}H NMR (500 MHz, $CDCl_3$) δ = 8.49 (d, J = 8.5 Hz, 6H), 8.47 (d, J = 8.5 Hz, 6H), 6.23 (m, 6H), 2.56 – 2.40 (m, 6H), 2.03 – 1.91(m, 12H), 1.73 – 1.61 (m, 6H). ^{13}C NMR (125 MHz, $CDCl_3$) δ 162.9, 162.6, 131.5, 130.9, 126.6, 126.3, 126.0, 54.1, 30.1, 25.9. HRMS-ESI for **NDI-Δ**; Calcd for $C_{60}H_{42}N_6O_{12}$: m/z = 1039.2939 $[M + H]^+$; Found: 1039.2928 $[M + H]^+$.

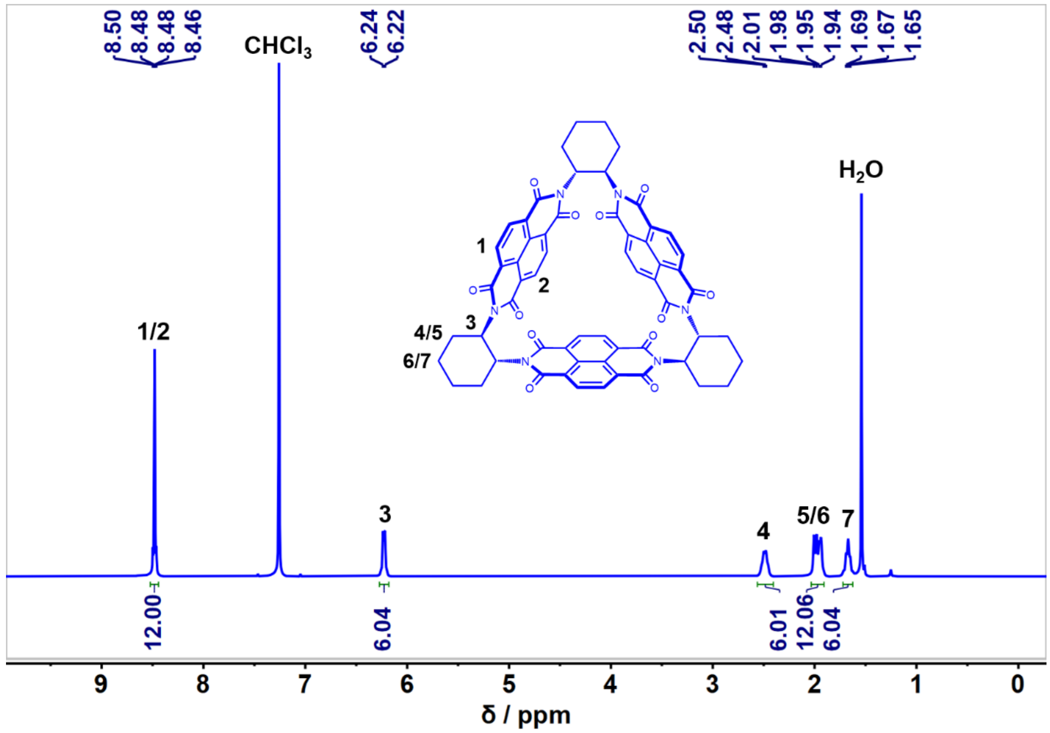

Supplementary Figure 2. ^{1}H NMR Spectrum (500 MHz, $CDCl_3$, 298 K) of **NDI-Δ**

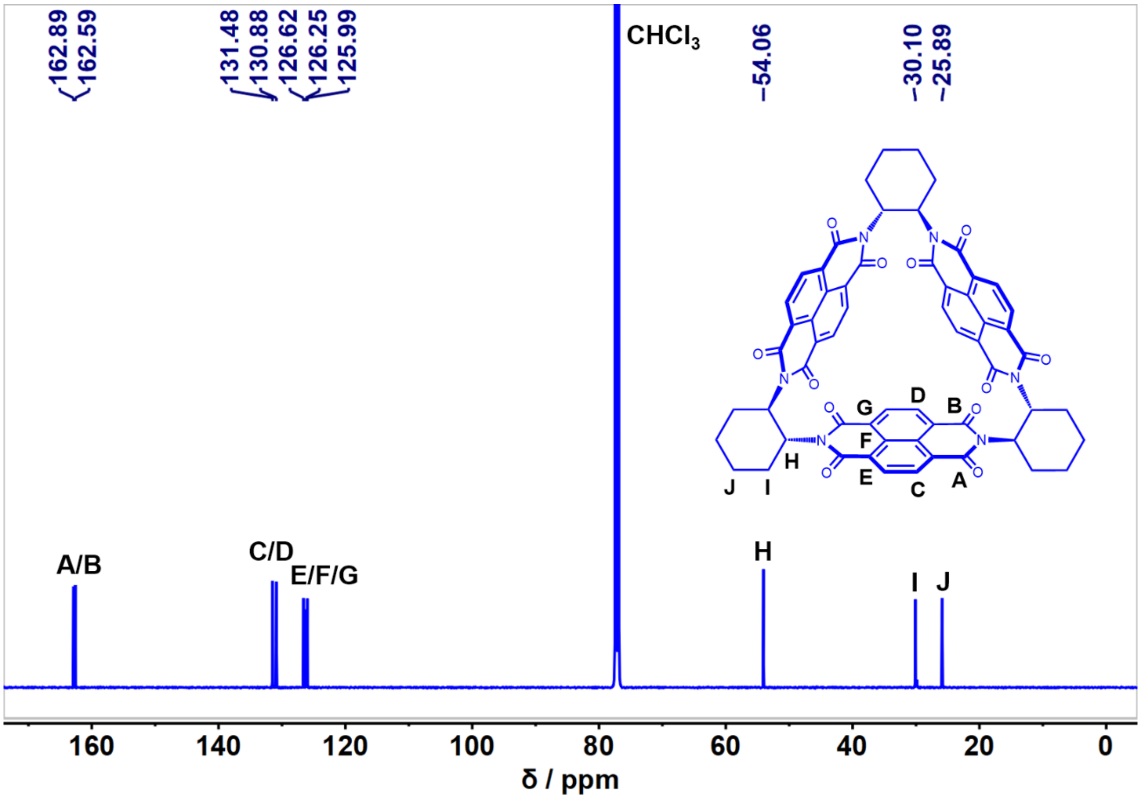

Supplementary Figure 3. ^{13}C NMR Spectrum (125 MHz, $CDCl_3$, 298 K) of **NDI-Δ**

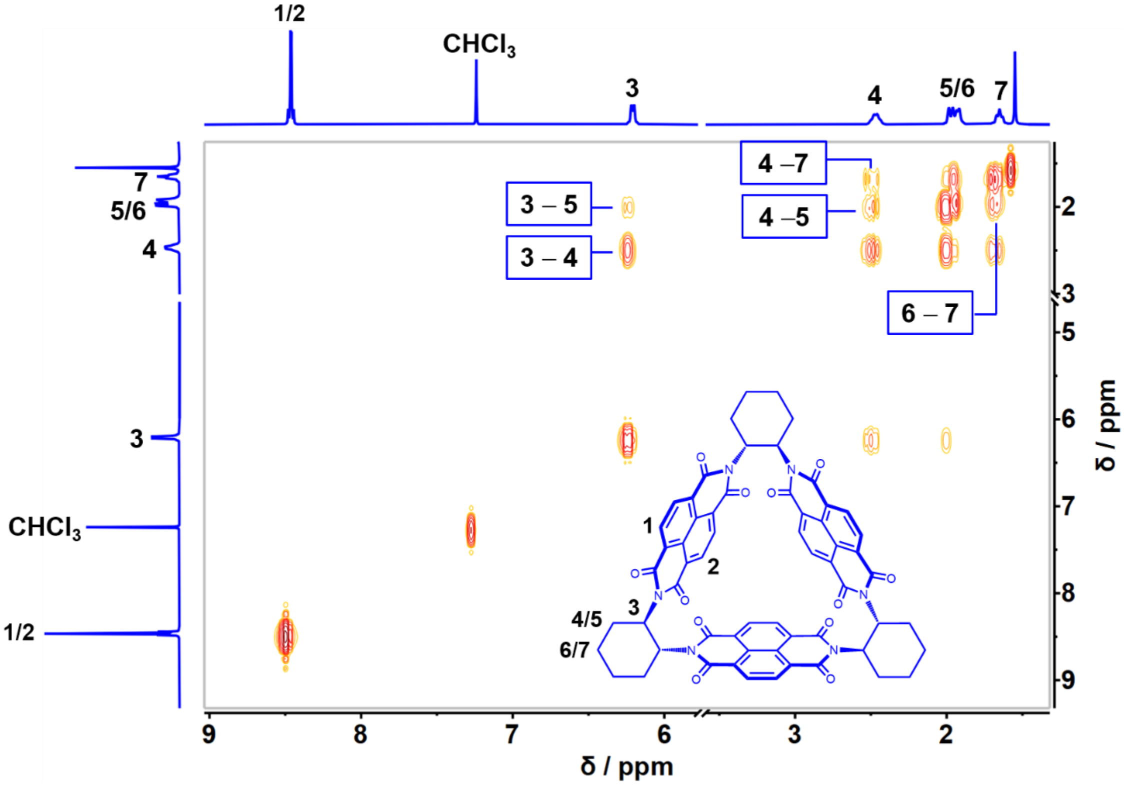

Supplementary Figure 4. Annotated 2D ^{1}H–^{1}H COSY NMR spectrum (500 MHz, $CDCl_3$, 298 K) of **NDI-Δ**

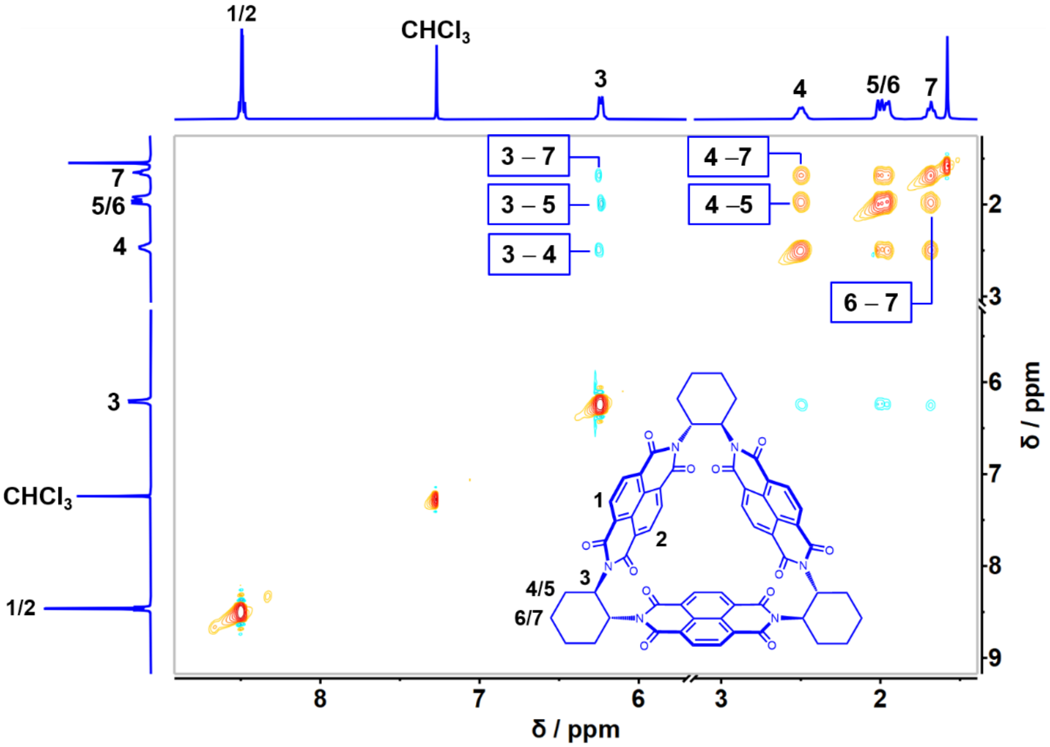

Supplementary Figure 5. Annotated 2D ^{1}H–^{1}H ROESY NMR spectrum (500 MHz, $CDCl_3$, 298 K) of **NDI-Δ**

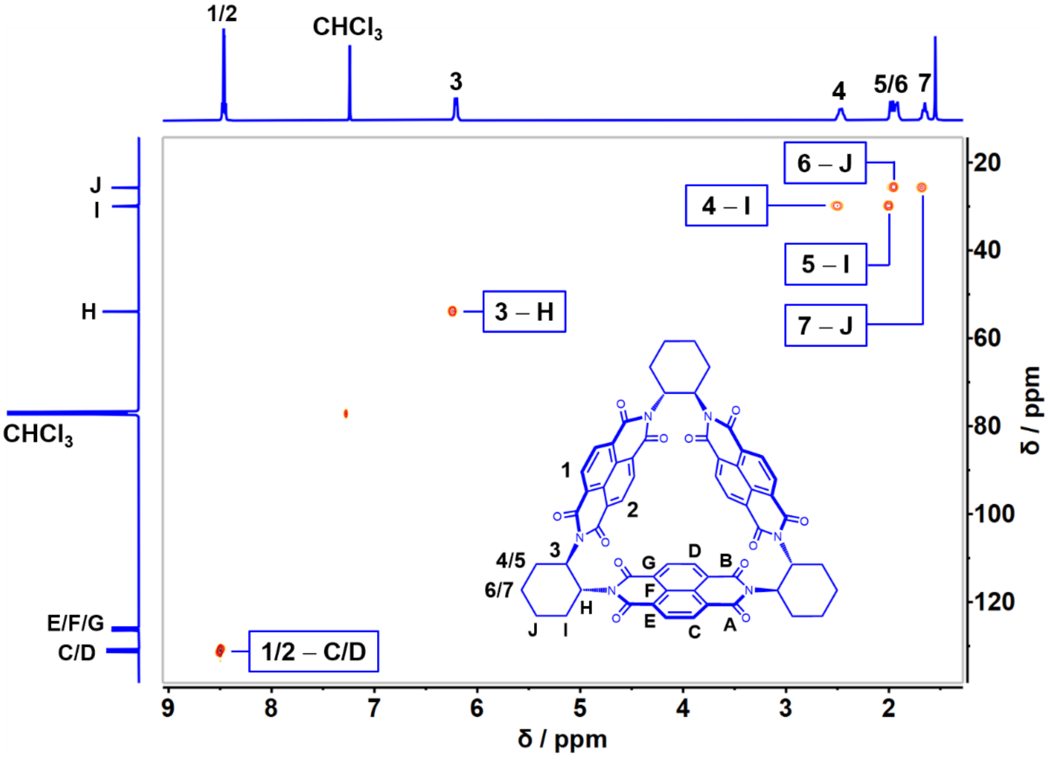

Supplementary Figure 6. Annotated ^{1}H–^{13}C Heteronuclear Single Quantum Coherence (HSQC) NMR spectrum (500 MHz, $CDCl_3$, 298 K) of **NDI-Δ**

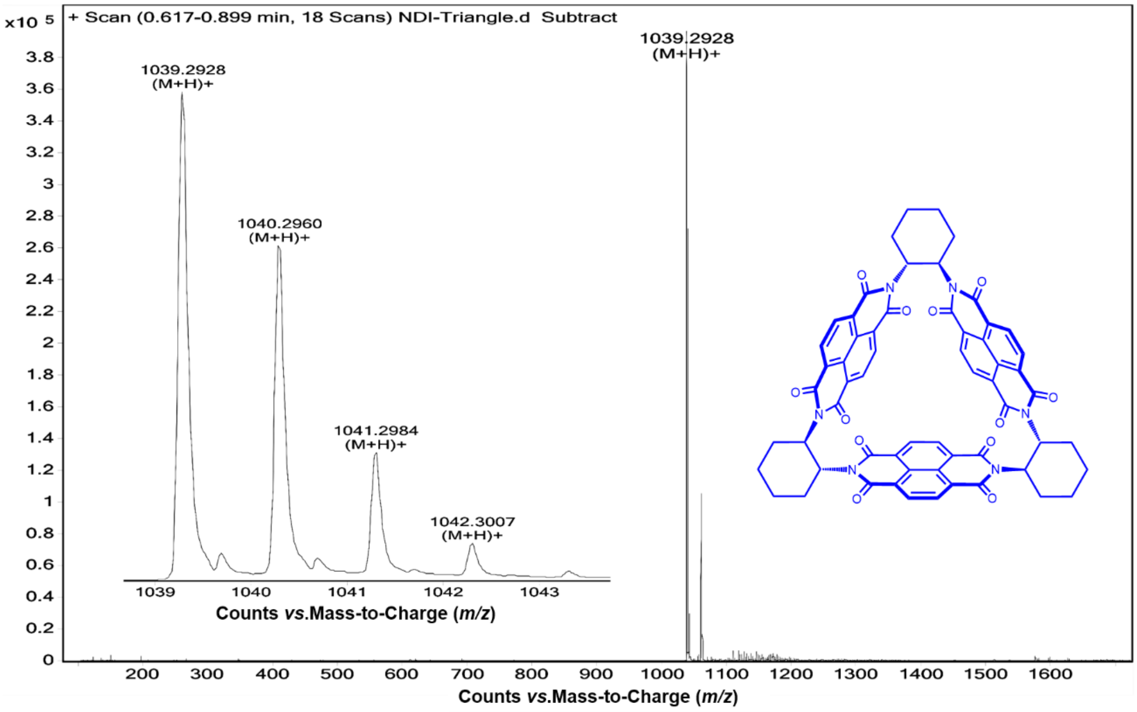

Supplementary Figure 7. High resolution mass spectrum (HRMS) of **NDI-Δ**

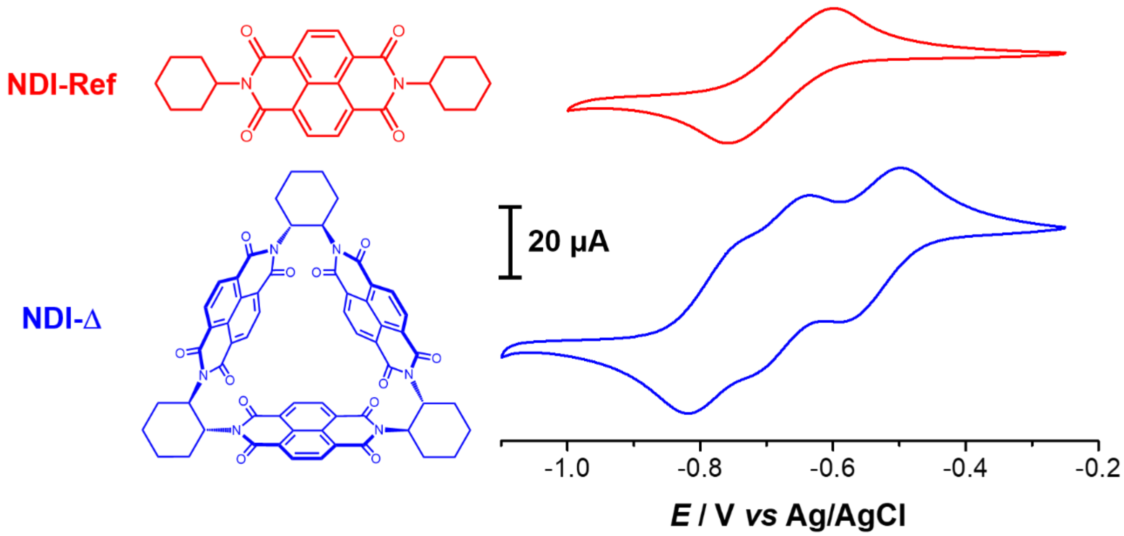

Supplementary Figure 8. Cyclic voltammograms (CVs) of an NDI reference compound (**NDI-Ref**, red curve) and **NDI-Δ** (blue curve) recorded using a glassy carbon electrode. The CV of **NDI-Ref** reveals a reversible redox wave with half-wave potential at –675 mV. The CV of **NDI-Δ** shows three sequential and distinct reversible redox waves with half-wave potentials at –531, –675 and –786 mV, respectively. The observation indicates the existence of electronic communication between the NDI units in the **NDI-Δ** molecule.

(2) Preparation of the Coronene and NDI-Δ Co-Crystals (CNCs)

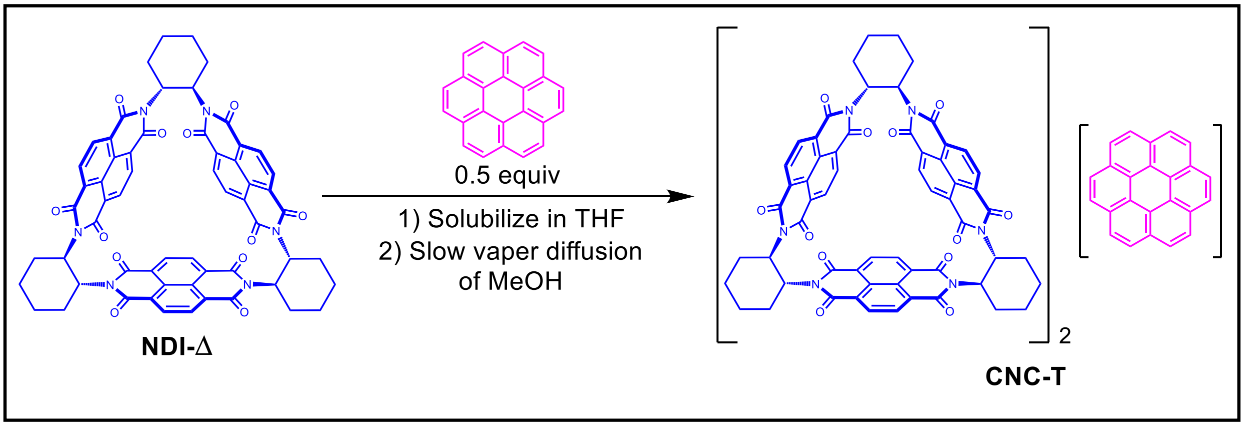

Supplementary Figure 9. Preparation of co-crystal **CNC-T**

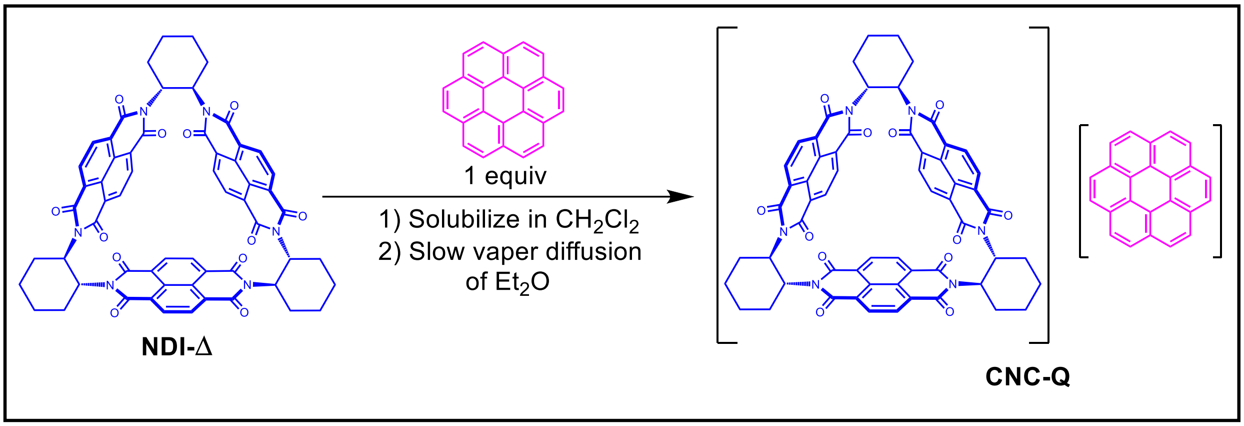

Supplementary Figure 10. Preparation of co-crystal **CNC-Q**

Supplementary Note 2. Crystallographic Characterization and Calculations

All crystallographic data are available free of charge from the Cambridge Crystallographic Data Centre (CCDC) via www.ccdc.cam.ac.uk/data_request/cif.

(1) NDI-Δ

(a) Methods. The **NDI-Δ** crystals, suitable for single crystal X-ray crystallography, were prepared by slow vapor diffusion. Detailed processes were performed as follows. **NDI-Δ** (1.0 mg, 1.0 µmol) was first dissolved in chlorobenzene (2 mL). After filtration of the solution with a 0.22-µm syringe filter, the filtrate was transferred into several 1-mL tubes, which were placed inside a 20-mL vial containing MeOH (3 mL). When MeOH diffused into the solution slowly, high quality colorless needle crystals of **NDI-Δ** were obtained after one week. A suitable crystal was selected and the crystal was mounted on a MITIGEN holder with Paratone oil on a XtaLAB Synergy, Single source at home/near, HyPix diffractometer. The crystal was kept at 100.01(10) K during data collection. Using Olex2,[1] the structure was solved with the ShelXT structure solution program[2] using Intrinsic Phasing and refined with the XL refinement package[3] using Least Squares minimization.

(b) Refinement details. Distance restraints were imposed on the disordered solvent molecules. The

enhanced rigid-bond restraint (SHELX keyword RIGU), as well as restraints on similar amplitudes separated by less than 1.7 Å were applied to the disordered solvent molecules.[4] Restraints of similar amplitudes were separated by less than 1.7 Å.

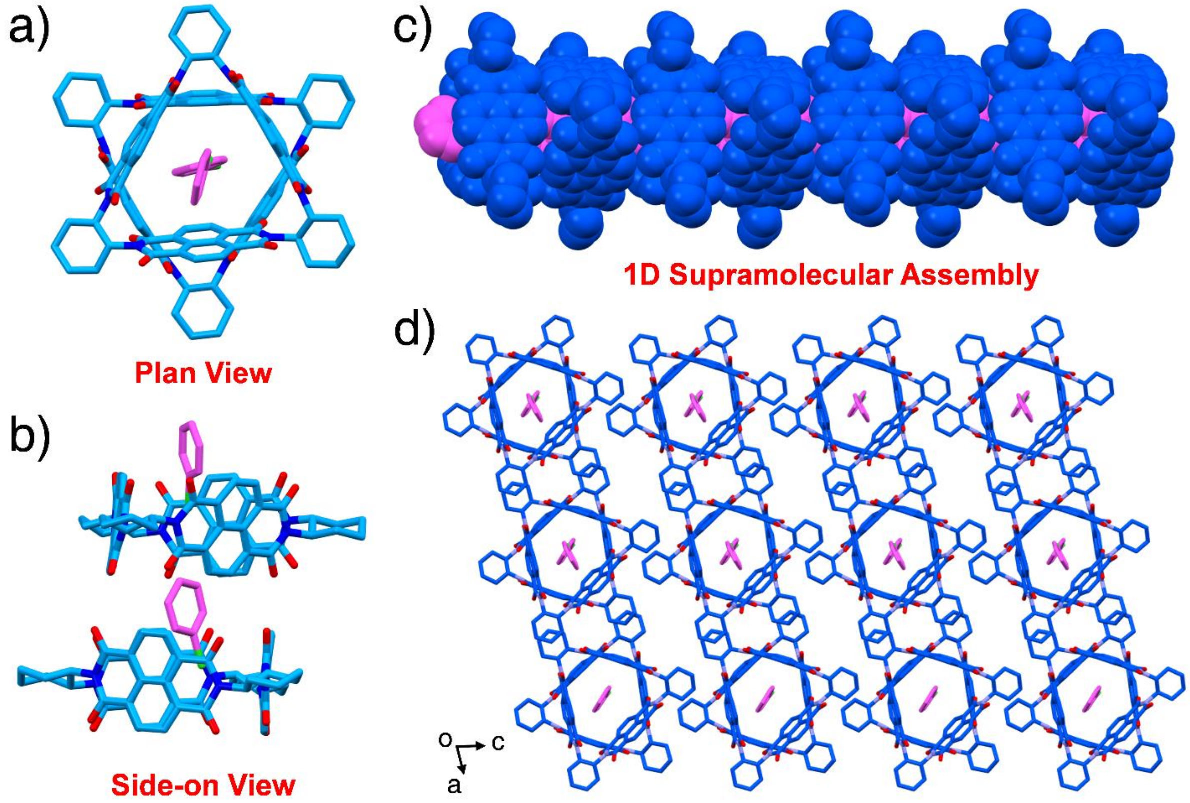

Supplementary Figure 11. Solid-state superstructures of **NDI-Δ** crystals. (a, b) Capped-sticks representations demonstrating that the PhCl molecule can be included in the **NDI-Δ** stacking column. (c) The 1D superstructure of **NDI-Δ** column extending along the *b*-axis. (d) Solid-state superstructure of **NDI-Δ** showing the layer-by-layer stacking.

(2) COR

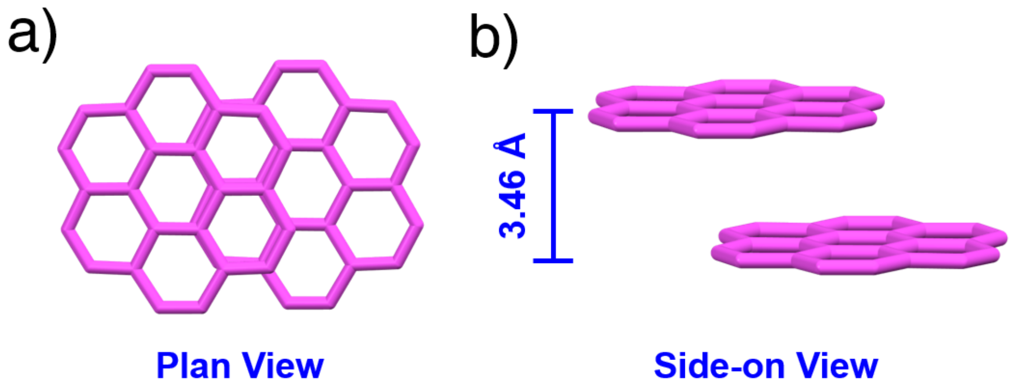

Supplementary Figure 12. (a) Plan view and (b) side-on view of solid-state superstructures of **COR**. The data was taken from CCDC number of 1129883.

(3) CNC-T

(a) Methods. High quality **CNC-T** co-crystals suitable for single crystal X-ray crystallography, were also prepared by slow vapor diffusion. Details of the experiments carried out are as follows. **NDI-Δ** (10.4 mg, 10 μmol) and **COR** (1.5 mg, 5 μmol) were first of all dissolved in THF (10 mL). After filtration of the solution with a 0.22-μm syringe filter, the filtrate was transferred into several 1-mL tubes, which were placed in a 20-mL vial containing MeOH (3 mL). With MeOH diffusing slowly into the THF solution, high quality red triangle-shaped co-crystals were obtained after one week. A suitable crystal was selected and it was mounted on a MITIGEN holder with Paratone oil on a XtaLAB Synergy, Single source, HyPix diffractometer. The crystal was kept at 100.01(10) K during data collection. Using Olex2,[1] the structure was solved with the ShelXT structure solution program[2] using Intrinsic Phasing and refined with the XL refinement package[3] using Least Squares minimization.

(b) Refinement details. Restraints on similar amplitudes separated by less than 1.7 Å were applied globally.

(c) Solvent treatment details. The solvent masking procedure as implemented in Olex2 was used to remove the electronic contribution of solvent molecules from the refinement. As the exact solvent content is not known, only the atoms used in the refinement model are reported in the formula here. Total solvent accessible volume / cell = 3829.8 Å^3 [28.2%] Total electron count / cell = 826.6.

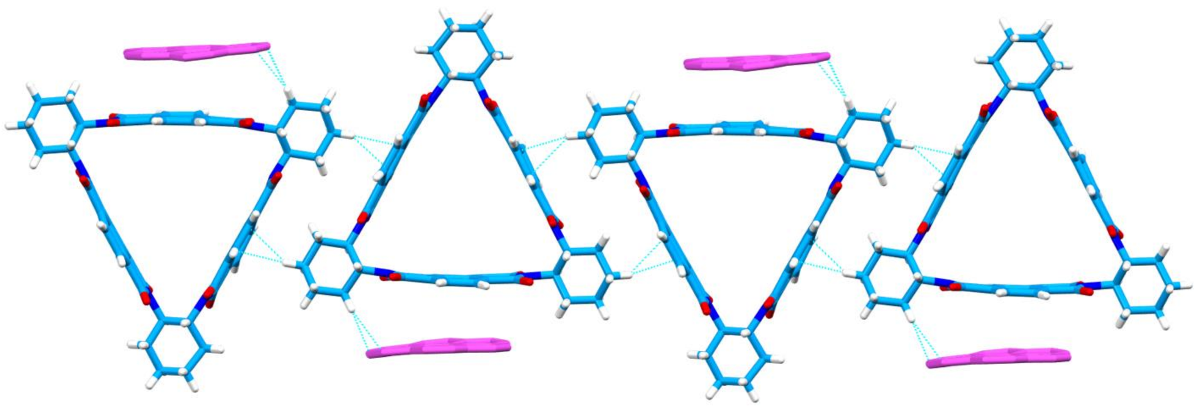

Supplementary Figure 13. Solid-state superstructure of **CNC-T** illustrating the intermolecular [C–H···π] interactions. The [C–H···π] interactions between the cyclohexano hydrogen atoms in **NDI-Δ** and the π-plane of **COR** range from 2.60 to 2.88 Å. The [C–H···π] interaction distances between the cyclohexano hydrogen atoms and the NDI plane in **NDI-Δ** contact with distances of 2.69 to 2.90 Å.

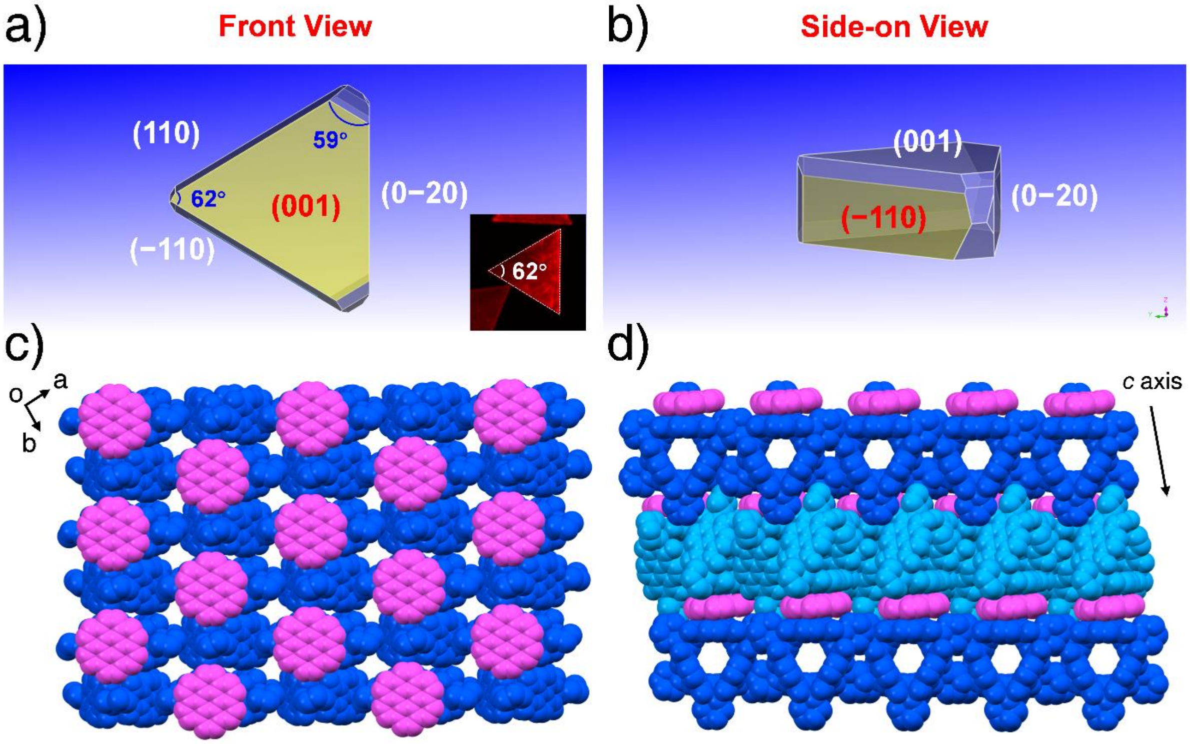

Supplementary Figure 14. Simulated crystal growth morphology of **CNC-T** performed by Material Studio software. (a) The (001) growth face in the simulated morphology and (c) the superstructure of **CNC-T** in the (001) lattice plane. (b) The (−110) growth face in the simulated morphology and (d) the superstructure of **CNC-T** in the (−110) lattice plane.

Supplementary Table 1. The Surface Attachment Energy of CNC-T Calculated by Material Studio

Surface	E_{att} (kcal / mol / unit cell)	Total facet area (Å^2 / unit cell)	% Total facet area
(001) and (00−1)	−121	2.25E+5	42.5
(110) and (−1−10)	−192	9.21E+4	16.0
(1−10) and (−110)	−192	9.21E+4	16.0
(020)	−202	4.91E+4	8.53
(0−20)	−202	4.91E+4	8.53
(11−1) and (−1−11)	−217	2.17E+4	4.21
(1−1−1) and (−111)	−217	2.17E+4	4.21

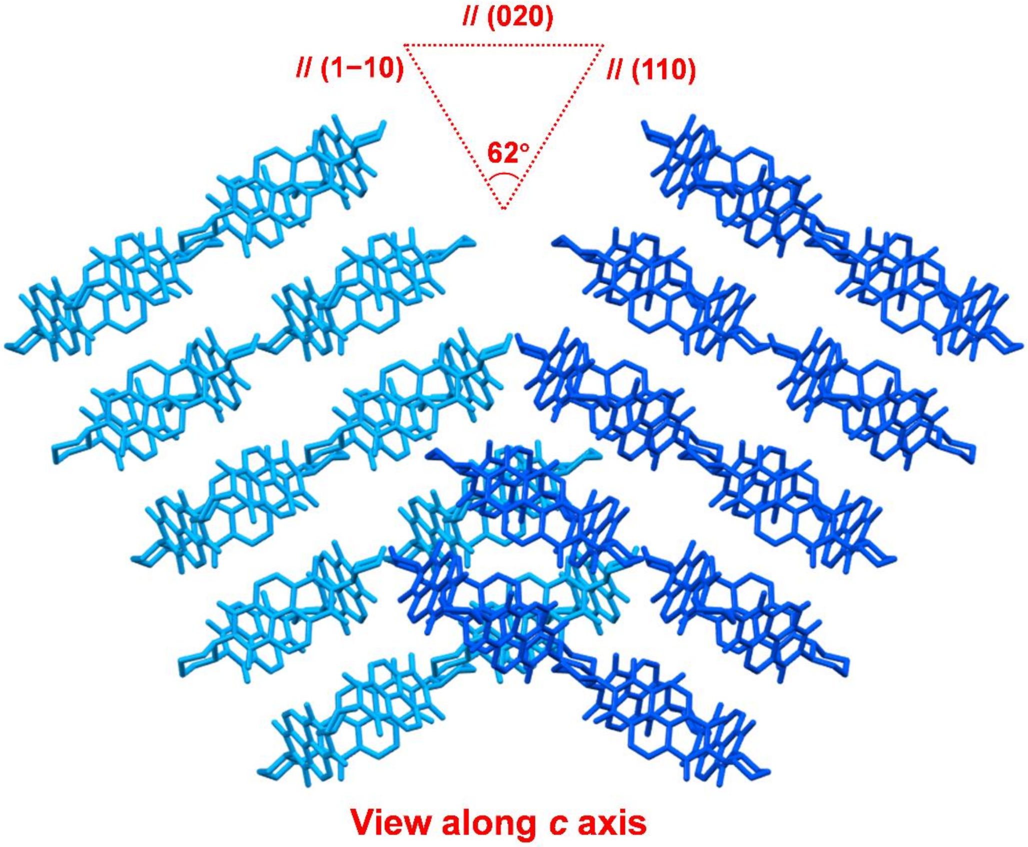

Supplementary Figure 15. Two adjacent layers in **CNC-T** superstructure view along the *c* axis. The adjacent layers demonstrate identical symmetry, and the **NDI-Δ** column stacking directions are parallel to (110) and (1−10) surface, respectively.

(4) CNC-Q

(a) Methods. **CNC-Q** co-crystals suitable for single crystal X-ray crystallography, were prepared by slow vapor diffusion. Details of the experiments carried out are as follows. **NDI-Δ** (10.4 mg, 10 μmol) and **COR** (3.0 mg, 10 μmol) were first of all dissolved in CH_2Cl_2 (10 mL). After filtration of the solution with a 0.22-μm syringe filter, the filtrate was transferred into several 1-mL tubes, which were placed in a 20-mL vial containing Et_2O (3 mL). With Et_2O diffusing slowly into the CH_2Cl_2 solution, high quality red quadrangular **CNC-Q** co-crystals were obtained after one week. A suitable crystal was selected and it was mounted on a MITIGEN holder with Paratone oil on a XtaLAB Synergy, Single source, HyPix diffractometer. The crystal was kept at 200.00(10) K during data collection. Using Olex2,[1] the structure was solved with the ShelXT structure solution program[2] using Intrinsic Phasing and refined with the XL refinement package[3] using Least Squares minimization.

(b) Refinement details. Two coronene molecules showed slight disorder and were refined the enhanced rigid-bond restraint (SHELX keyword RIGU).[4] The coronene labled C was also refined with restrained carbon–carbon distances.

(c) Solvent treatment details. The solvent masking procedure as implemented in Olex2 was used to remove the electronic contribution of solvent molecules from the refinement. As the exact solvent content is not known, only the atoms used in the refinement model are reported in the formula here. Total solvent accessible volume / cell = 2598.6 Å^3 [32.2%] Total electron count / cell = 655.6.

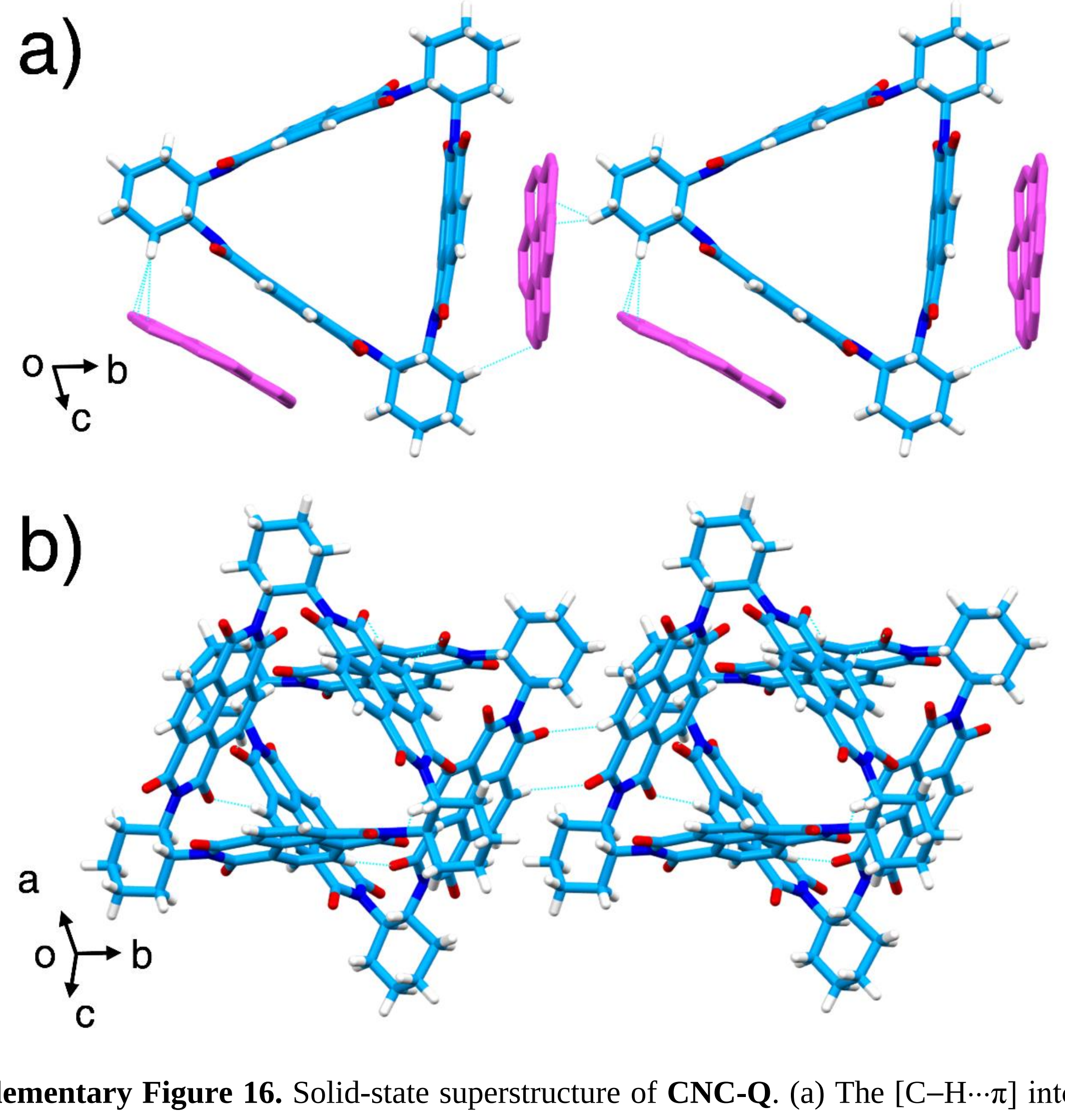

Supplementary Figure 16. Solid-state superstructure of **CNC-Q**. (a) The [C–H···π] interactions between **NDI-Δ** and **COR** with distances ranging from 2.66 to 2.90 Å. (b) The coaxial **NDI-Δ** dimer stack through multiple hydrogen bonds with distances of 2.22 to 2.57 Å. Two neighbouring **NDI-Δ** dimers contact with each other only through two hydrogen bonds with distances of 2.32 and 2.58 Å.

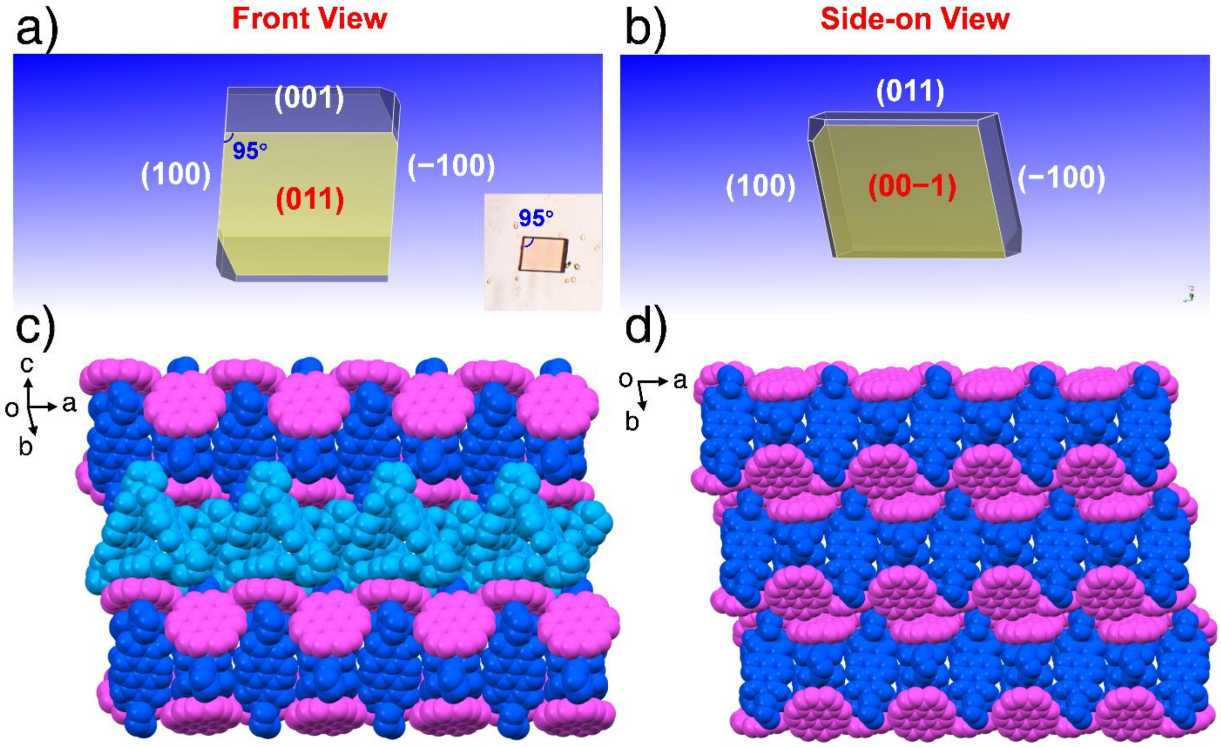

Supplementary Figure 17. Simulated crystal growth morphology of **CNC-Q** executed by Material Studio software. (a) The (011) growth face in the simulated morphology and (c) the superstructure of **CNC-Q** in the (011) lattice plane. (b) The (00−1) growth face in the simulated morphology and (d) the superstructure of **CNC-Q** in the (00−1) lattice plane.

Supplementary Table 2. The Calculated Surface Attachment Energy of CNC-Q by Material Studio

Surface	E_{att} (kcal / mol / unit cell)	Total facet area (Å^2 / unit cell)	% Total facet area
(001)	−64.3	2.24E+4	17.3
(00−1)	−64.3	2.24E+4	17.3
(011)	−66.4	2.17E+4	16.8
(0−1−1)	−66.4	2.17E+04	16.8
(100)	−81.0	1.73E+4	13.4
(−100)	−81.0	1.73E+4	13.4

Supplementary Table 3. Crystallographic Data for NDI-Δ, CNC-T and CNC-Q

**Complex**	**NDI-Δ**	**CNC-T**	**CNC-Q**
Empirical formula	$C_{84}H_{62}Cl_4N_6O_{12}$	$C_{146}H_{104}N_{12}O_{26}$	$C_{84}H_{54}N_6O_{12}$
Formula weight	1489.19	2442.41	1339.33
T / K	100.01(10)	100.01(10)	200.00(10)
Crystal system	triclinic	monoclinic	triclinic
Space group	$P1$	$C2$	$P1$
a / Å	15.1584(7)	16.6166(18)	15.9445(3)
b / Å	15.6864(4)	27.553(2)	19.3096(3)
c / Å	16.1257(4)	30.158(3)	27.2086(4)
α / °	77.763(2)	90	77.2130(10)
β / °	73.882(3)	99.838(10)	89.5070(10)
γ / °	74.958(3)	90	81.0050(10)
V / Å^3	3516.8(2)	13605(2)	8065.9(2)
Z	2	4	4
ρ_{calcd} / g cm^{-3}	1.406	1.192	1.103
μ / mm^{-1}	2.118	0.681	0.609
F (000)	1544	5088	2784
goodness-of-fit on F^2	1.048	0.947	1.012
R_1 [$I > 2\sigma(I)$]	0.0664	0.0877	0.0594
wR_2 [all data]	0.1795	0.2516	0.1724
CCDC No.	2004158	2004159	2004160

Supplementary Note 3. Powder X-Ray Diffraction Analysis

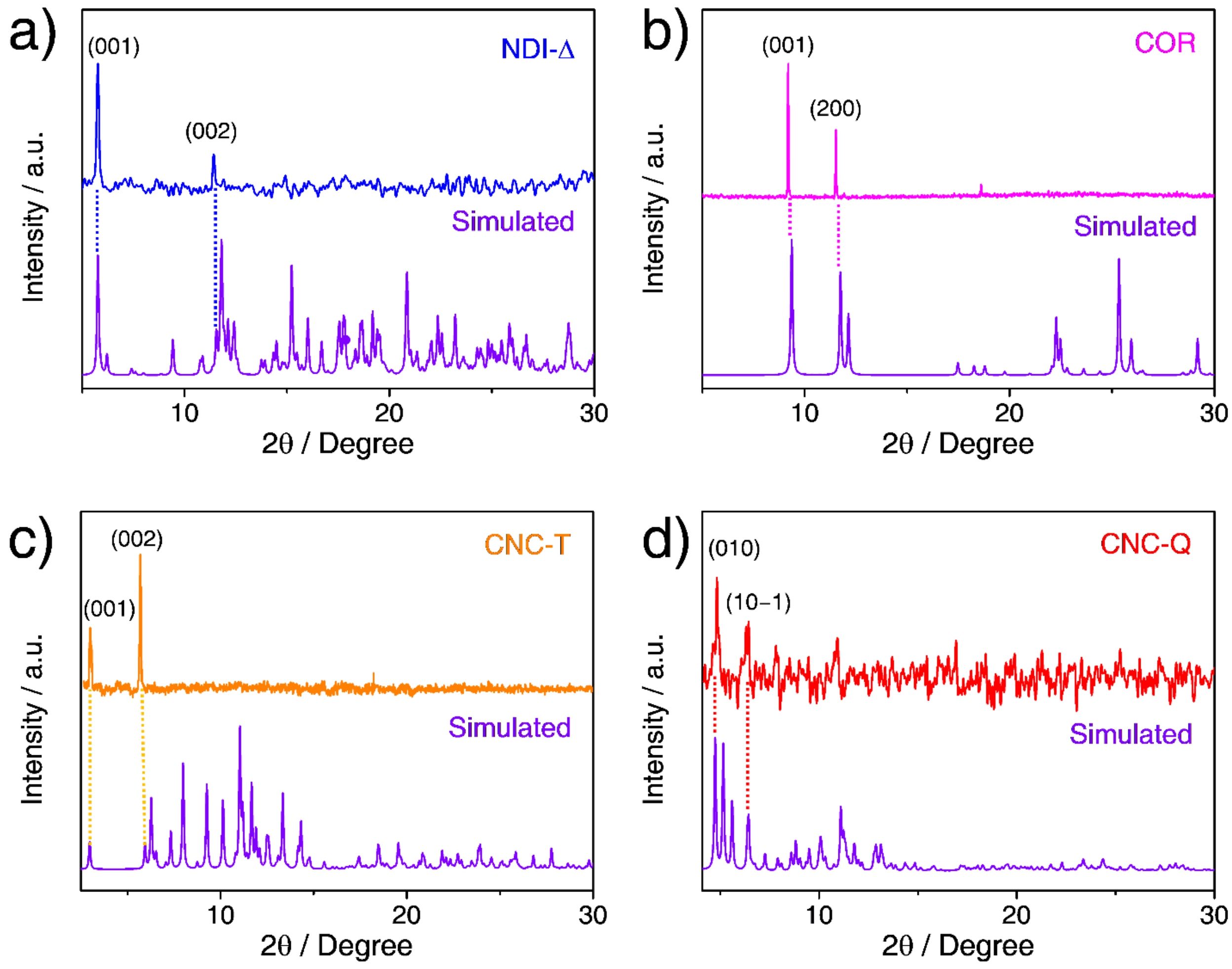

Supplementary Figure 18. Powder X-ray diffraction data of (a) **NDI-Δ**, (b) **COR**, (c) **CNC-T** and (d) **CNC-Q**, accompanied with their corresponding simulated powder patterns carried out using X-ray crystallographic data. The single-crystal data for **COR** was taken from CCDC number of 1129883. In comparison with the powder X-ray diffraction patterns of individual **NDI-Δ** and **COR** crystals, those for the co-crystals display new diffraction peaks, illustrating the formation of new crystal systems. Moreover, the experimental diffraction data of **CNC-T** and **CNC-Q** are in accordance with their simulated patterns, implying the selective growth of the two co-crystals.

Supplementary Note 4. Photophysical Characterization

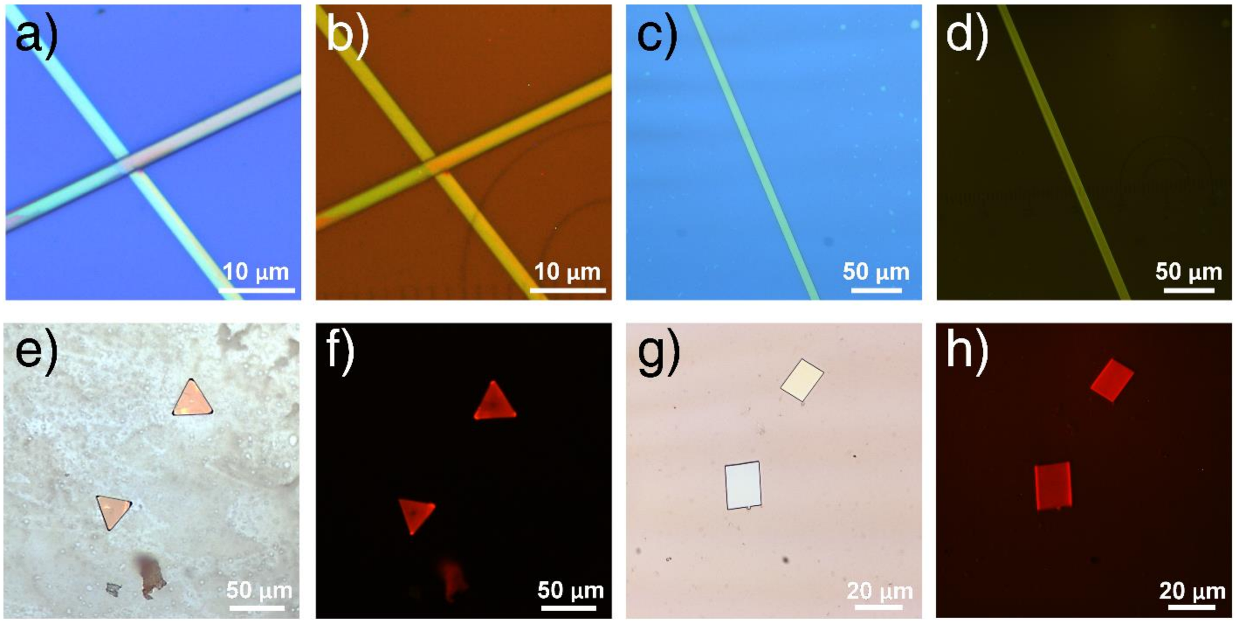

Supplementary Figure 19. Optical microscopy images and corresponding fluorescence microscopy images of (a, b) **NDI-Δ**, (c, d) **COR**, (e, f) **CNC-T** and (g, h) **CNC-Q**.

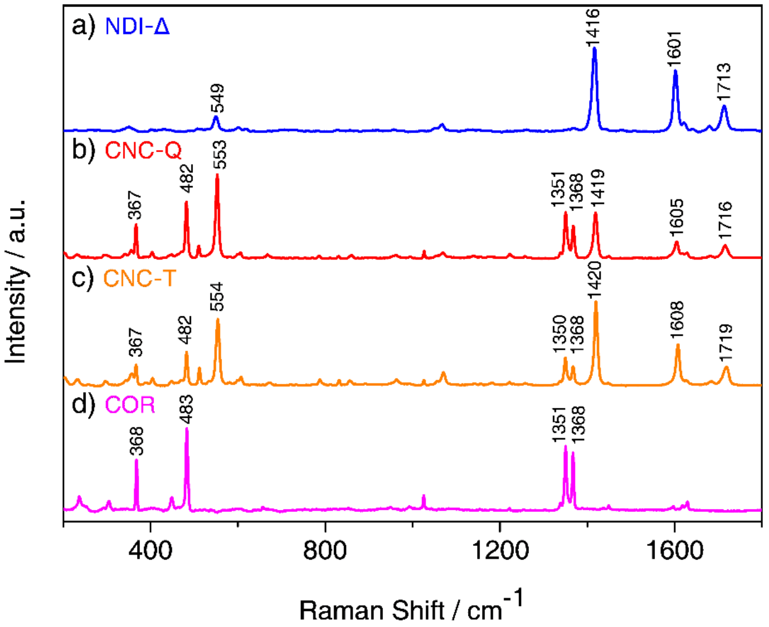

Supplementary Figure 20. Raman spectra of (a) **NDI-Δ**, (b) **CNC-Q**, (c) **CNC-T** and (d) **COR**, collected under excitation at 532, 785, 785, 785 nm, respectively. The spectra of **CNC-T** and **CNC-Q** are the combination of two individual components. They not only contain the naphthalene C=O stretching (1713 cm^{-1}), aromatic C=C and C–C stretching (1416, 1601 cm^{-1}) of **NDI-Δ**, but also includes the aromatic C–C stretching (1351, 1368 cm^{-1}) and C–C–C out-of-plane bending (368, 483 cm^{-1}) of **COR**, indicating the formation of two co-crystals.

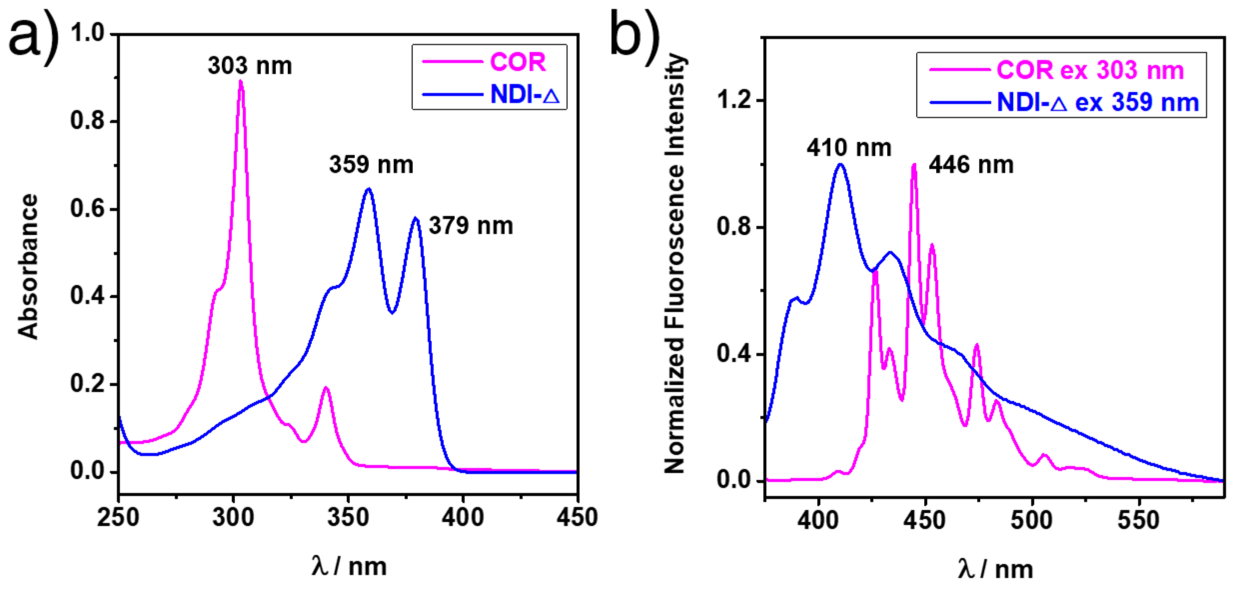

Supplementary Figure 21. The solution state (a) UV-Vis absorption and (b) fluorescence spectra of **NDI-Δ** (10 μM in CH_2Cl_2, 298 K) and **COR** (8 μM in CH_2Cl_2, 298 K).

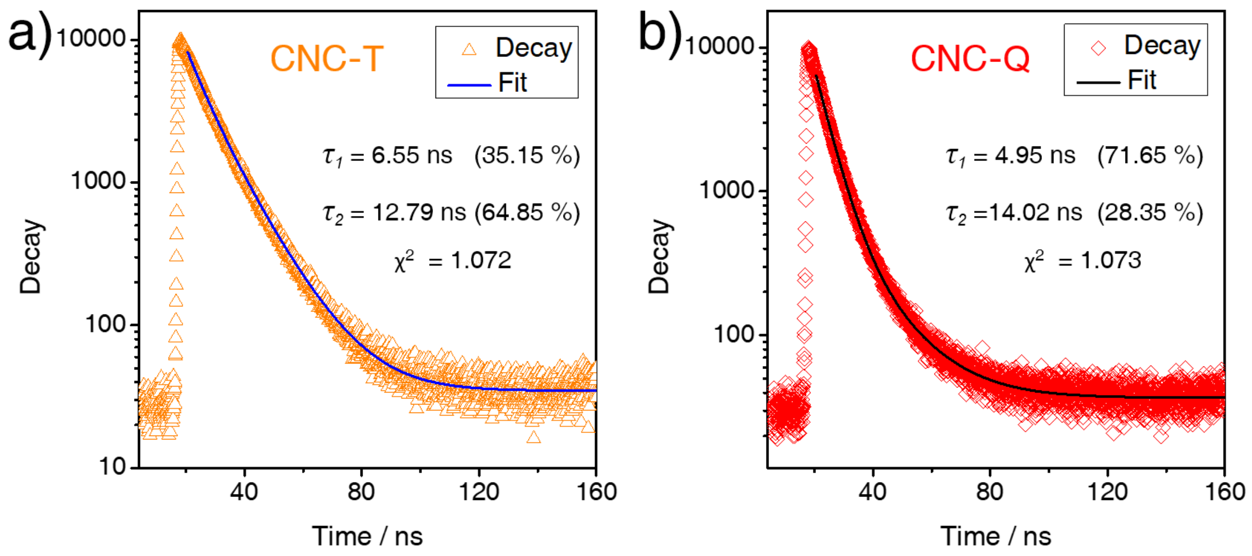

Supplementary Figure 22. The fluorescence decay curves of (a) **CNC-T** and (b) **CNC-Q** excited at 374 nm laser. Both **CNC-T** and **CNC-Q** afforded a double-exponential fluorescence decay curve with an average lifetime of 11.4 and 9.7 ns, respectively.

Supplementary Table 4. Photophysical Data for CNC-T and CNC-Q

	Φ_F (%)	τ_{avg} (ns)	k_r (s^{-1})	k_{nr} (s^{-1})
**CNC-T**	0.9	11.4	7.9×10^5	8.7×10^7
**CNC-Q**	2.2	9.7	2.3×10^6	1.0×10^8

Supplementary Note 5. Two-Photon Absorption Analysis

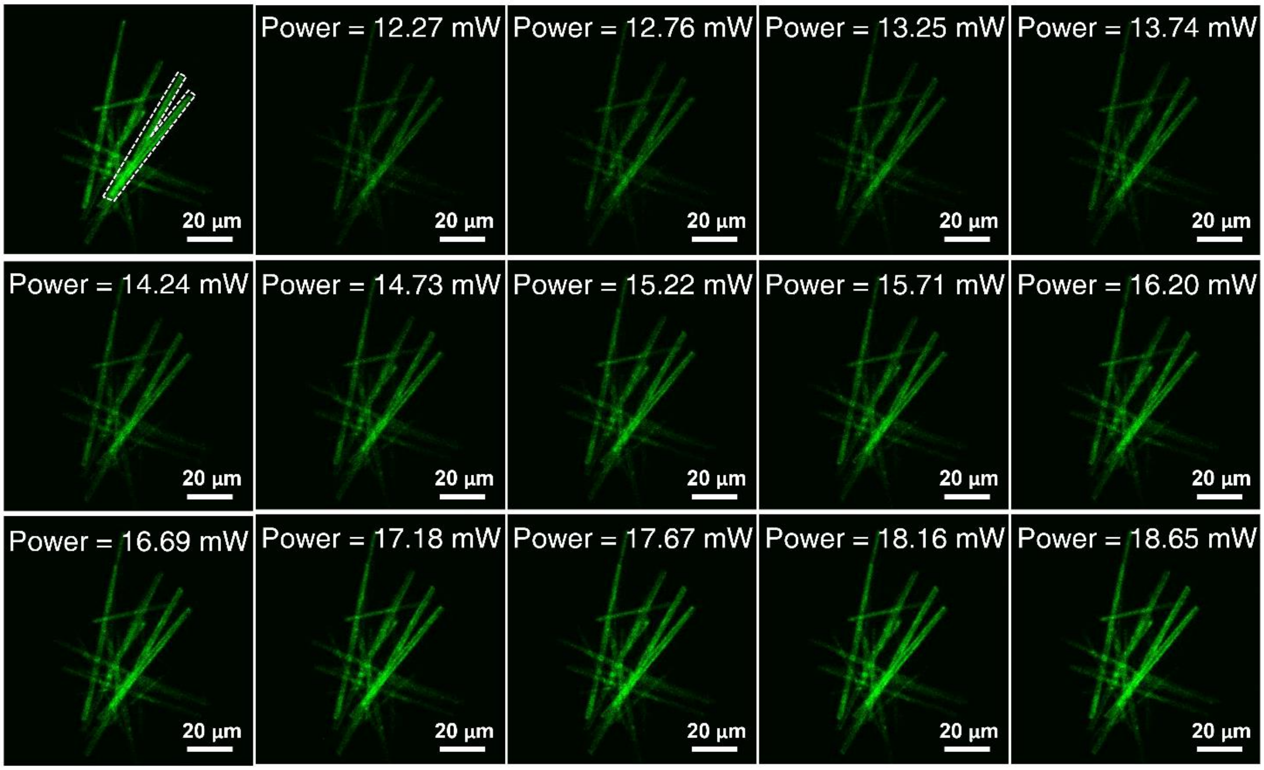

Supplementary Figure 23. The two-photon microscopy images of **NDI-Δ** excited at 740 nm with tunable laser powers.

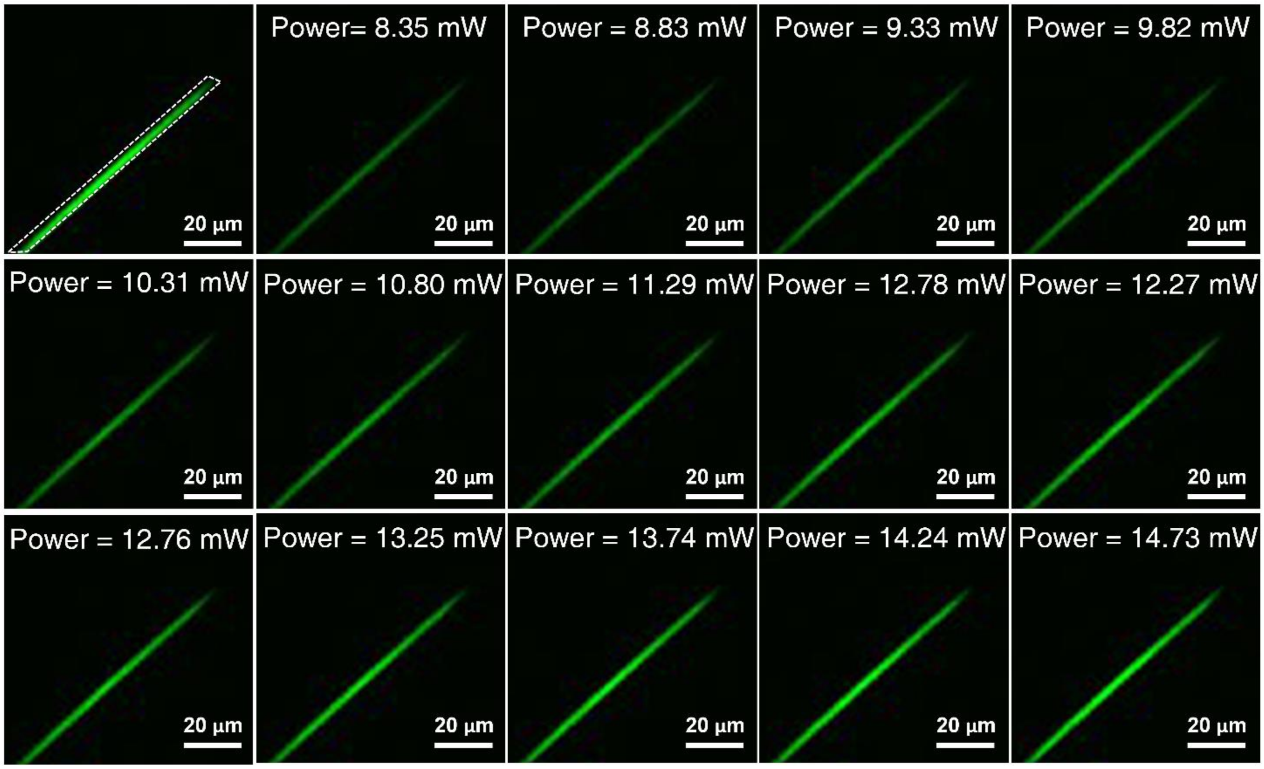

Supplementary Figure 24. The two-photon microscopy images of **COR** excited at 740 nm with tunable laser powers.

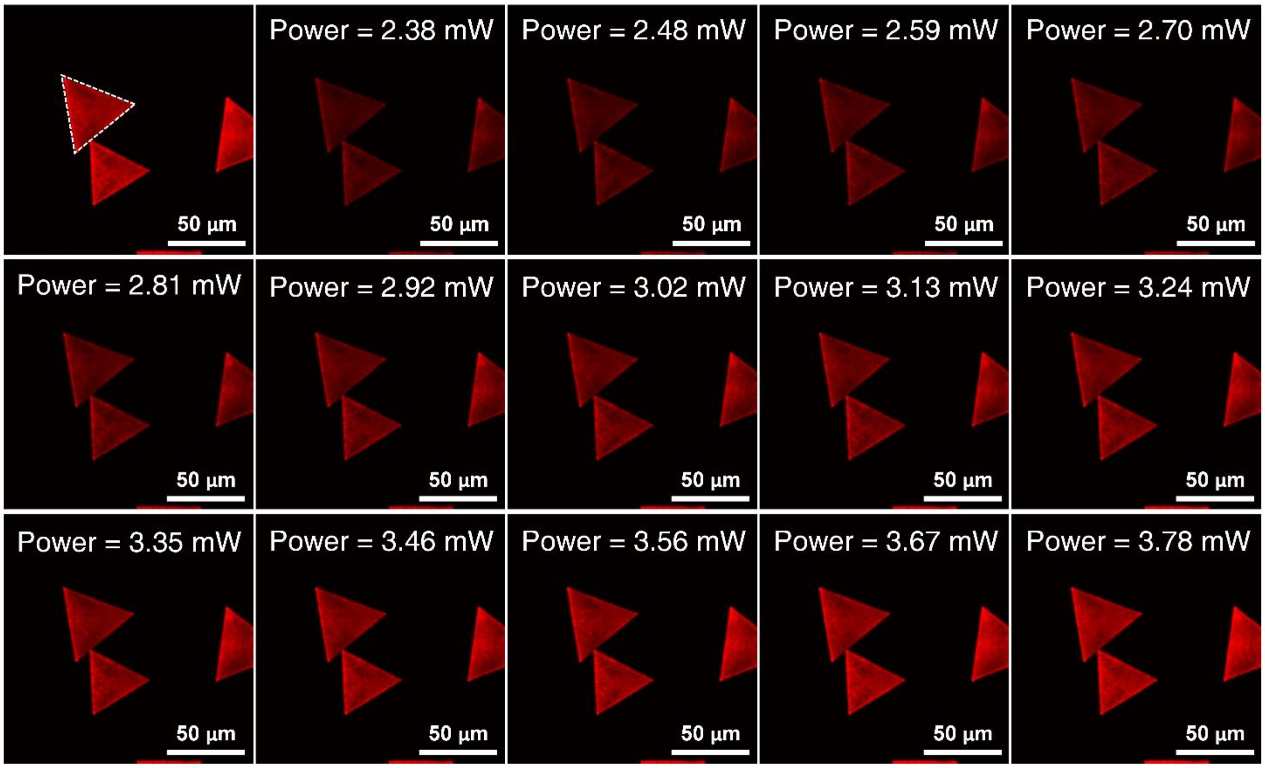

Supplementary Figure 25. The two-photon microscopy images of **CNC-T** excited at 1000 nm with tunable laser powers.

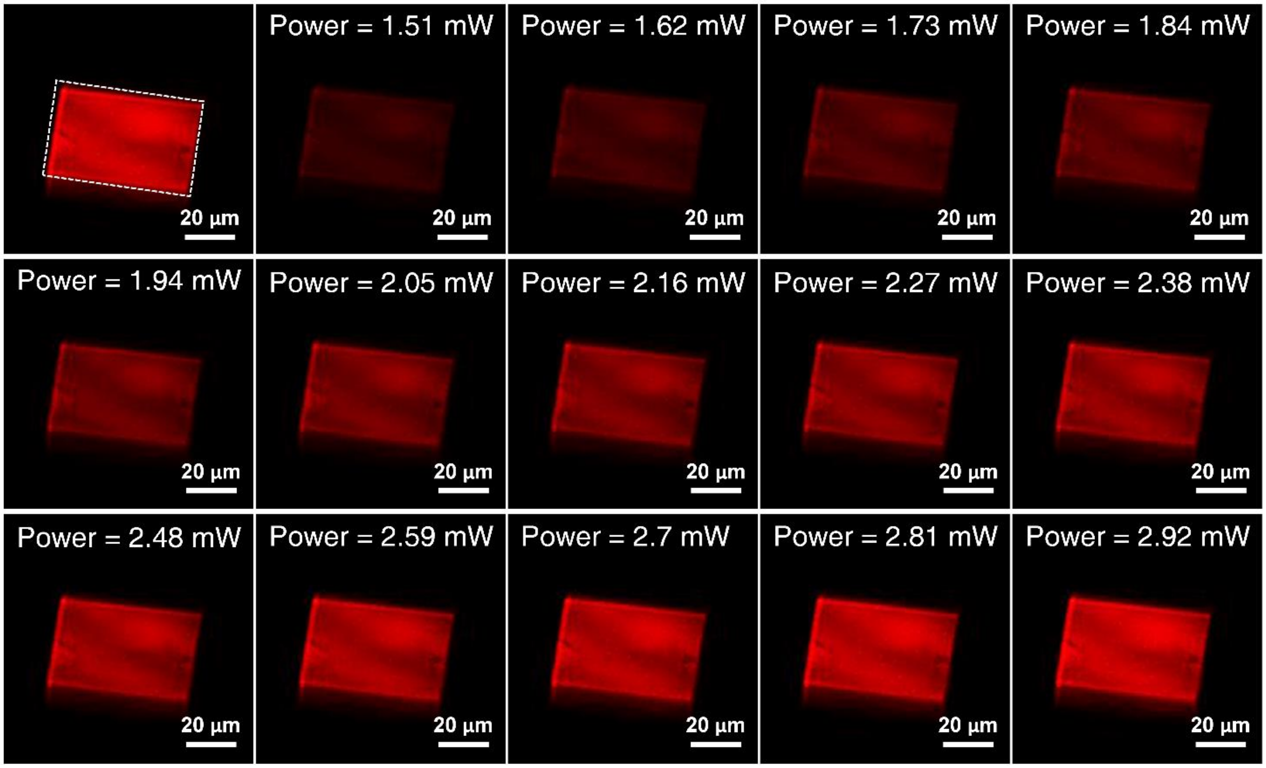

Supplementary Figure 26. The two-photon microscopy images of **CNC-Q** excited at 1000 nm with tunable laser powers.

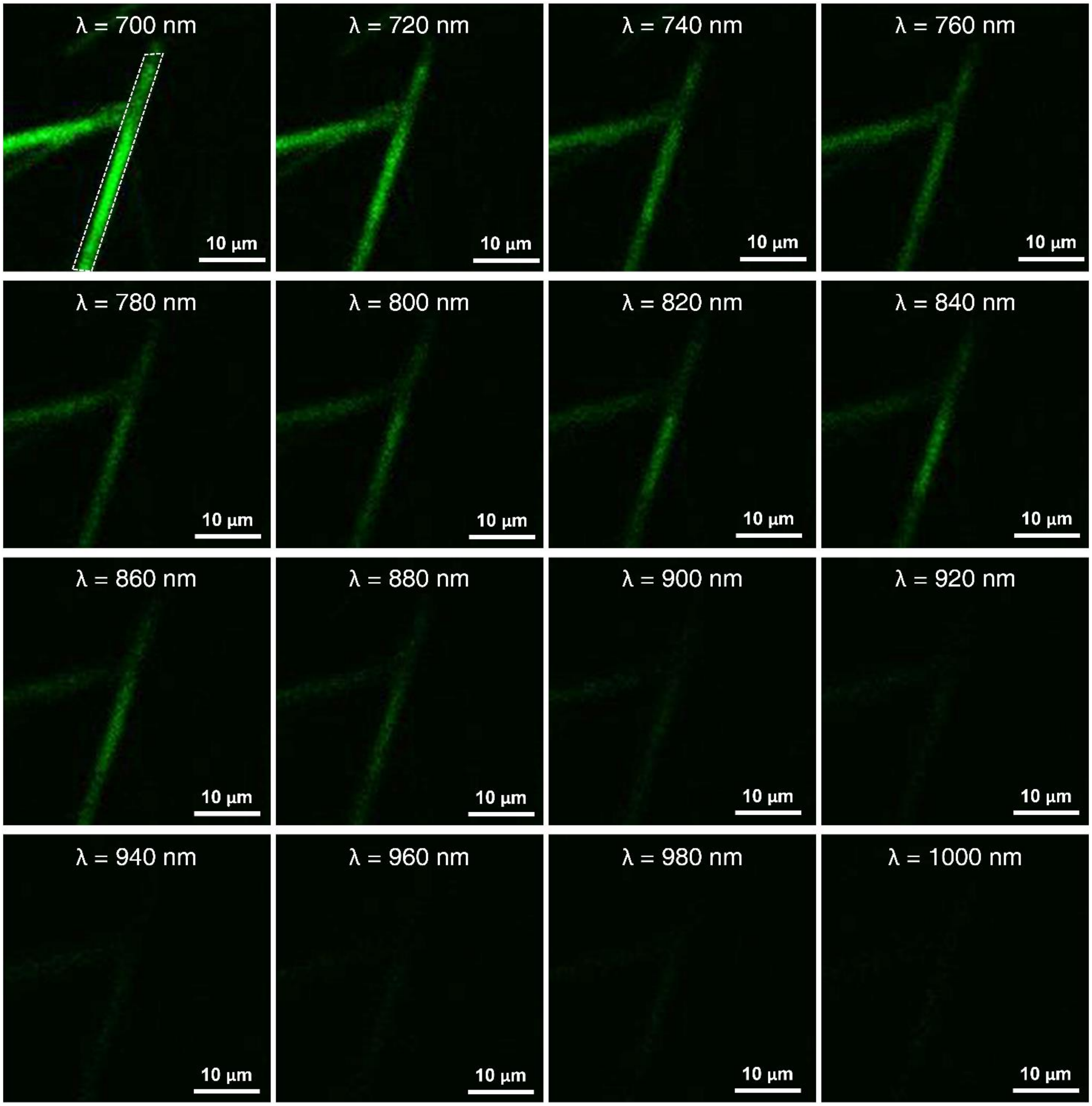

Supplementary Figure 27. The two-photon microscopy images of **NDI-Δ** excited at tunable excitation wavelengths from 700 to 1000 nm with a constant laser power of 13.3 mW. When excited at 700 nm laser, **NDI-Δ** crystals display the strongest upconversion fluorescence. With an increase of excitation wavelength, the intensity of upconversion fluorescence decreases, indicating the weak two-photon absorption. Notably, the crystals are hard to be observed at excitation wavelength longer than 900 nm.

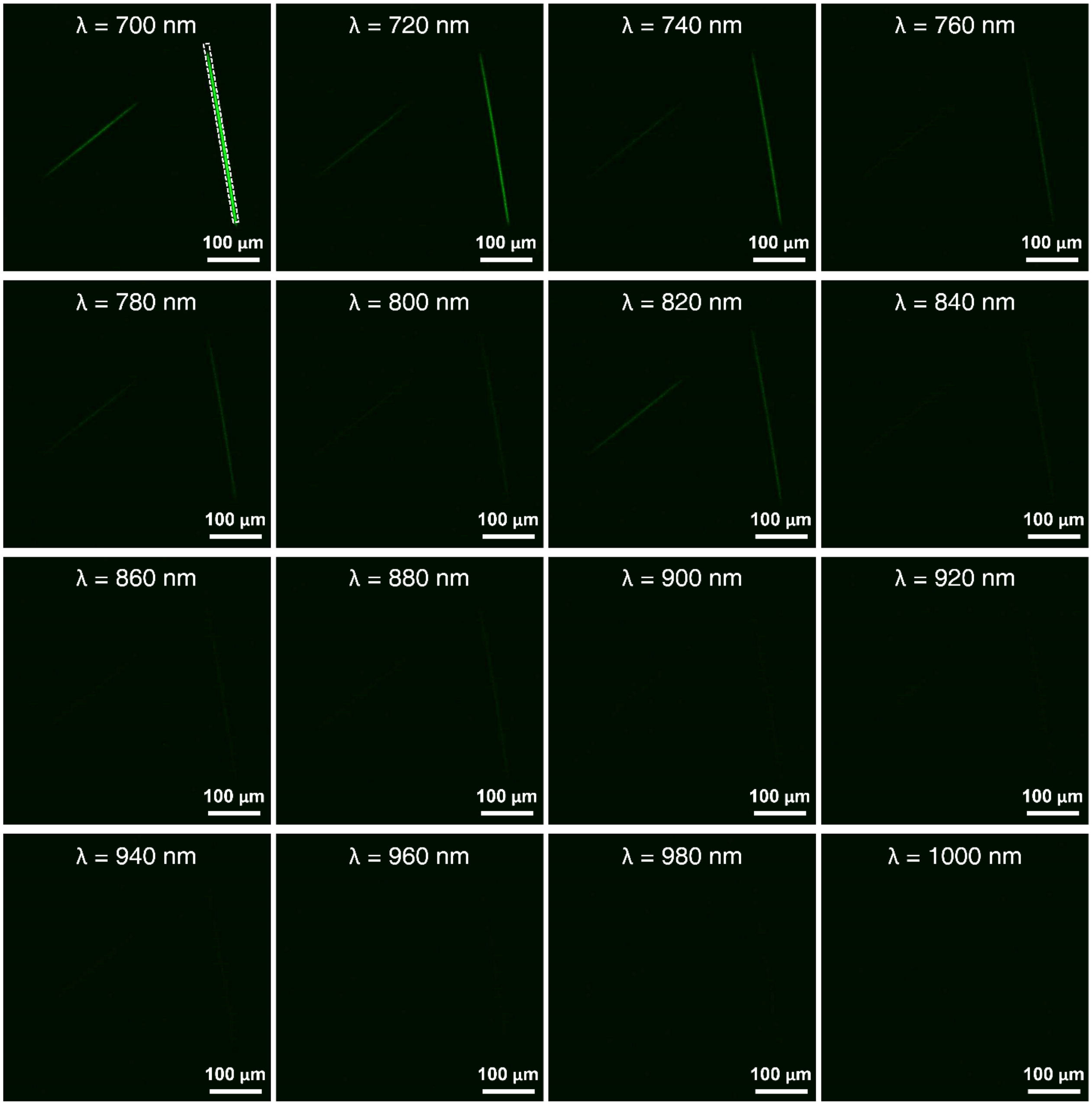

Supplementary Figure 28. The two-photon microscopy images of **COR** excited at tunable excitation wavelengths from 700 to 1000 nm with a constant laser power of 2.7 mW. When excited at 700 nm laser, **COR** crystals display the strongest two-photon excited fluorescence. With an increase of excitation wavelength, the intensity of upconversion fluorescence decreases, indicating the weak two-photon absorption. Notably, the crystals are hard to be observed at excitation wavelength longer than 840 nm.

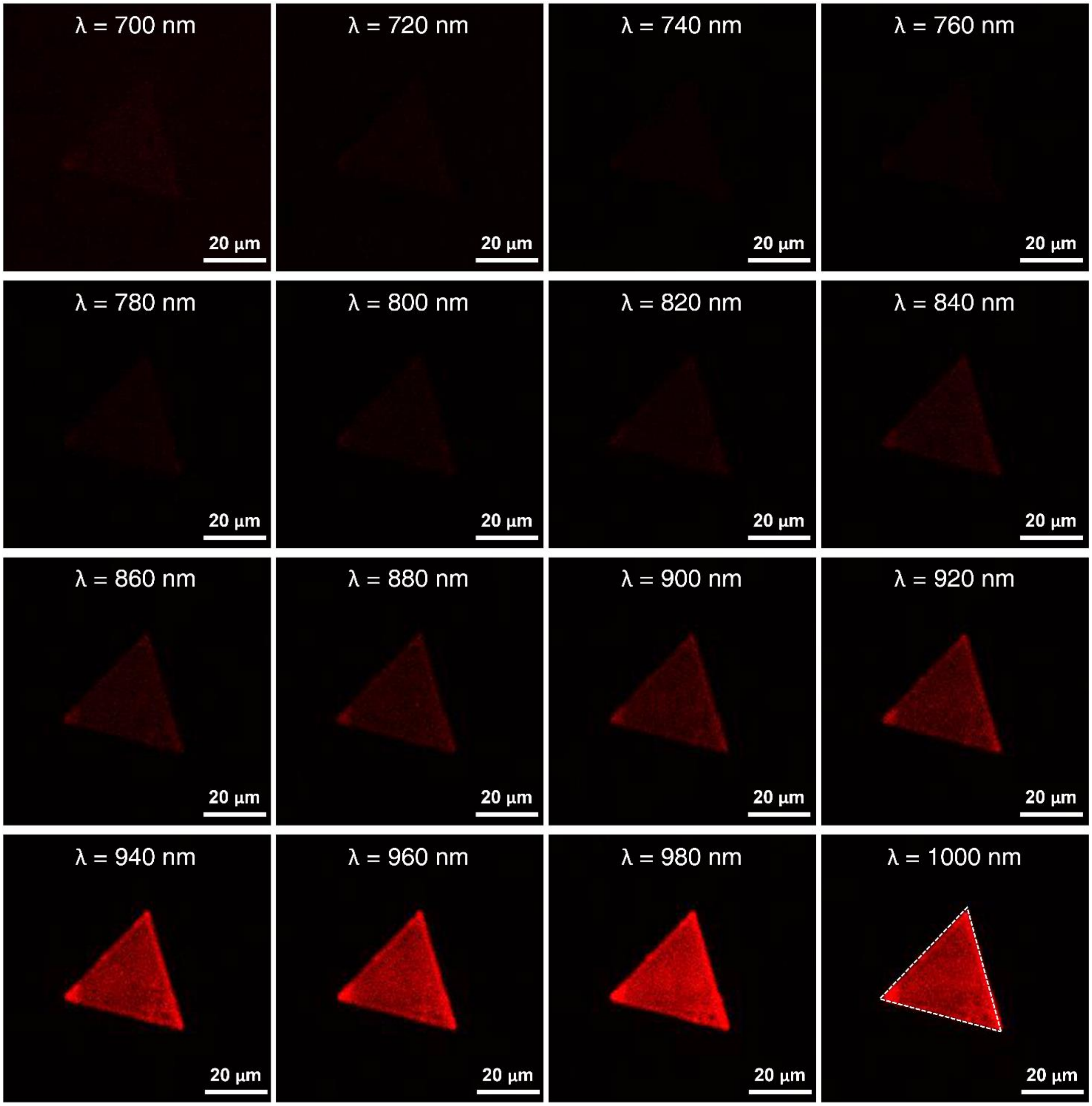

Supplementary Figure 29. The two-photon microscopy images of **CNC-T** excited at tunable excitation wavelengths from 700 to 1000 nm with a constant laser power of 2.7 mW. In sharp comparison with **NDI-Δ** and **COR**, **CNC-T** displays significant two-photon absorption in the region of 860 to 1000 nm. It exhibits the strongest two-photon excited fluorescence when excited at 980 nm laser, indicating that the two-photon absorption spectra of co-crystal is also red-shifted similar to those of one-photon absorption.

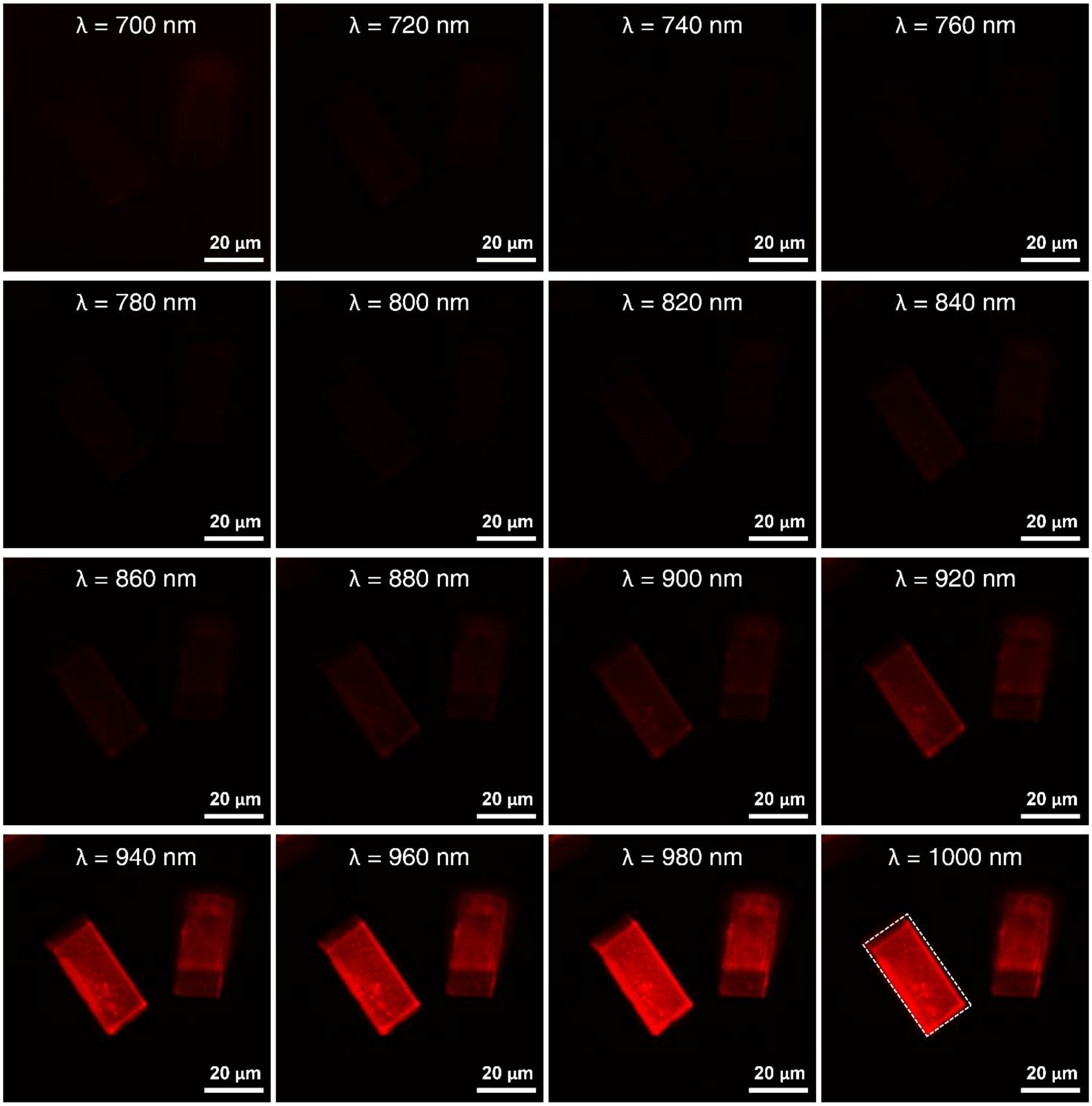

Supplementary Figure 30. The two-photon microscopy images of **CNC-Q** excited at tunable excitation wavelengths from 700 to 1000 nm with a constant laser power of 2.7 mW. Similar to **CNC-T**, **CNC-Q** displays significant two-photon absorption in the region of 880 to 1000 nm and exhibits the strongest two-photon excited fluorescence when excited at 980 nm laser. The two-photon spectrum of **CNC-Q** is red-shifted compared with those of **NDI-Δ** and **COR**.

Supplementary Note 6. Density Functional Theory Calculations

(1) Calculation of frontier molecular orbitals

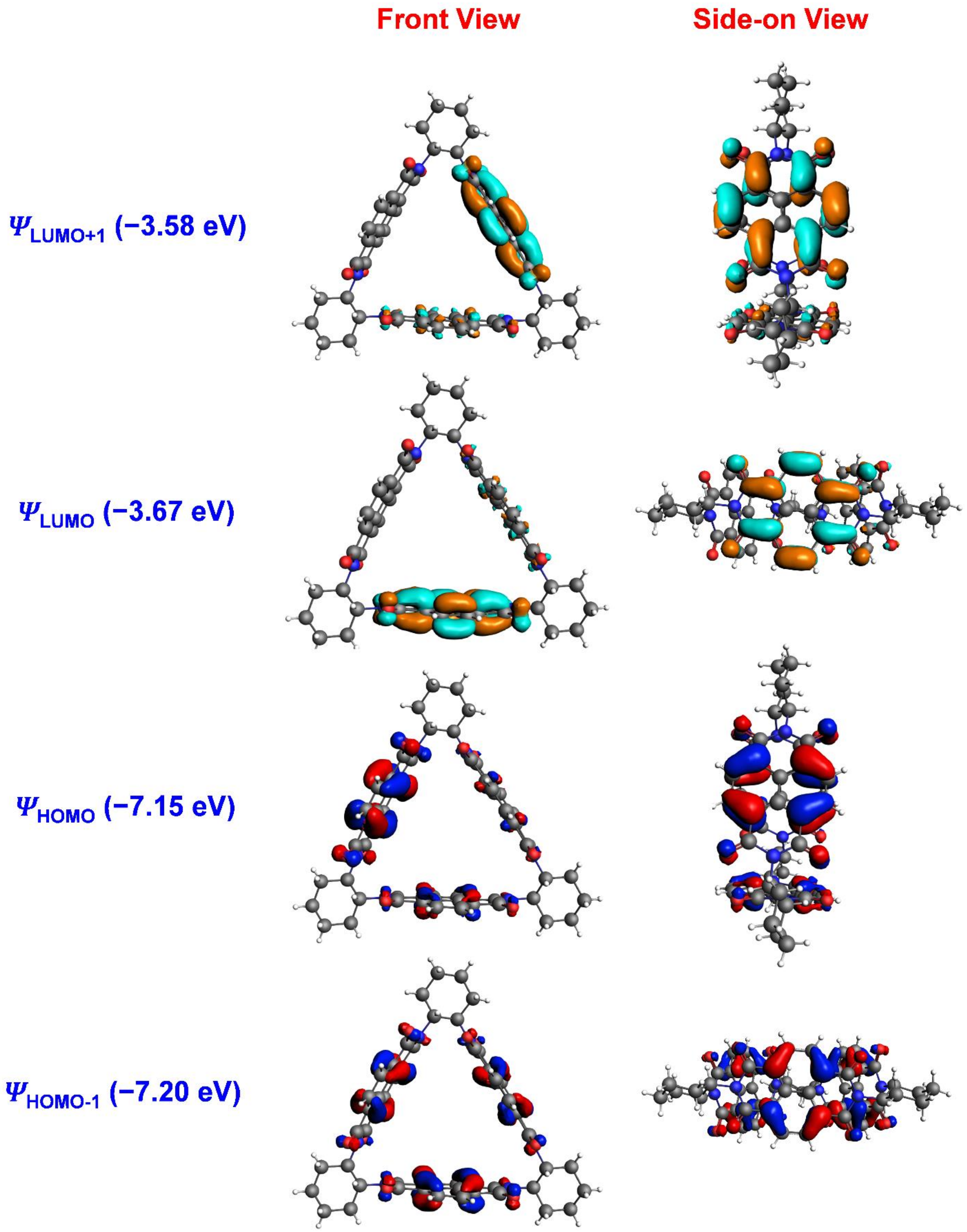

Supplementary Figure 31. Frontier molecular orbitals of **NDI-Δ**. The energy bandgap is calculated to be 3.47 eV.

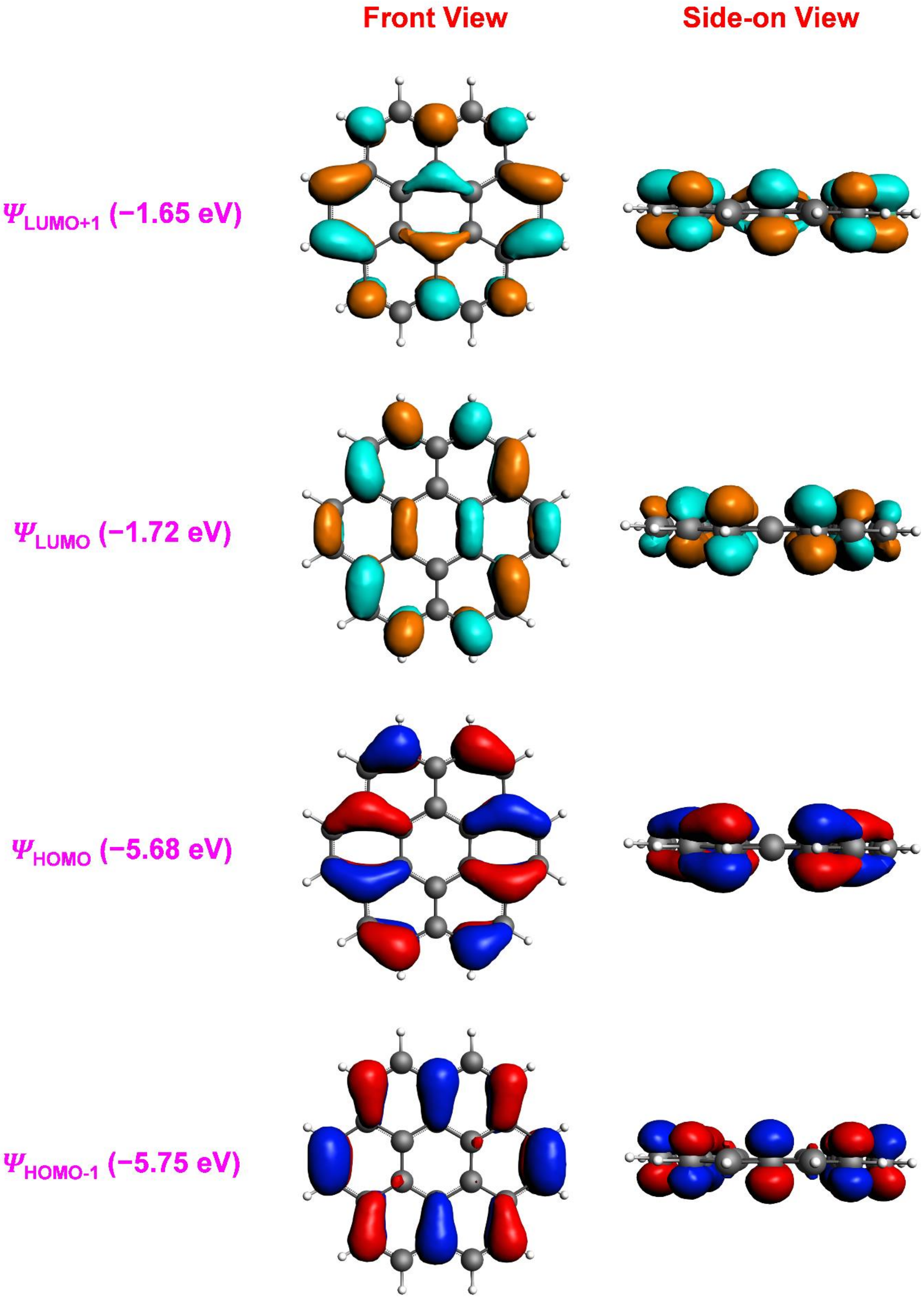

Supplementary Figure 32. Frontier molecular orbitals of **COR**. The energy bandgap is calculated to be 3.97 eV.

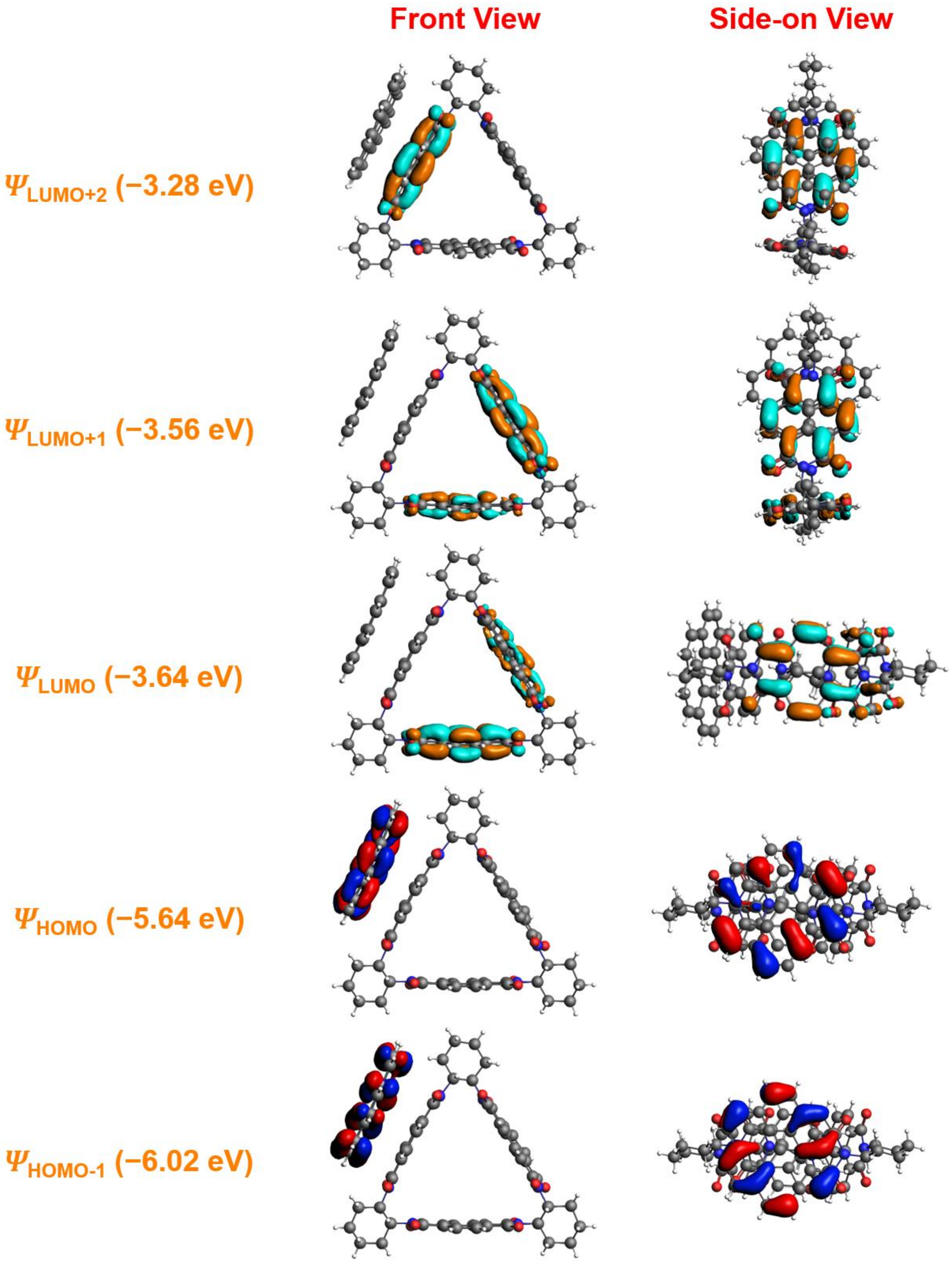

Supplementary Figure 33. Frontier molecular orbitals of **CNC-T**. The energy bandgap is calculated to be 2.00 eV.

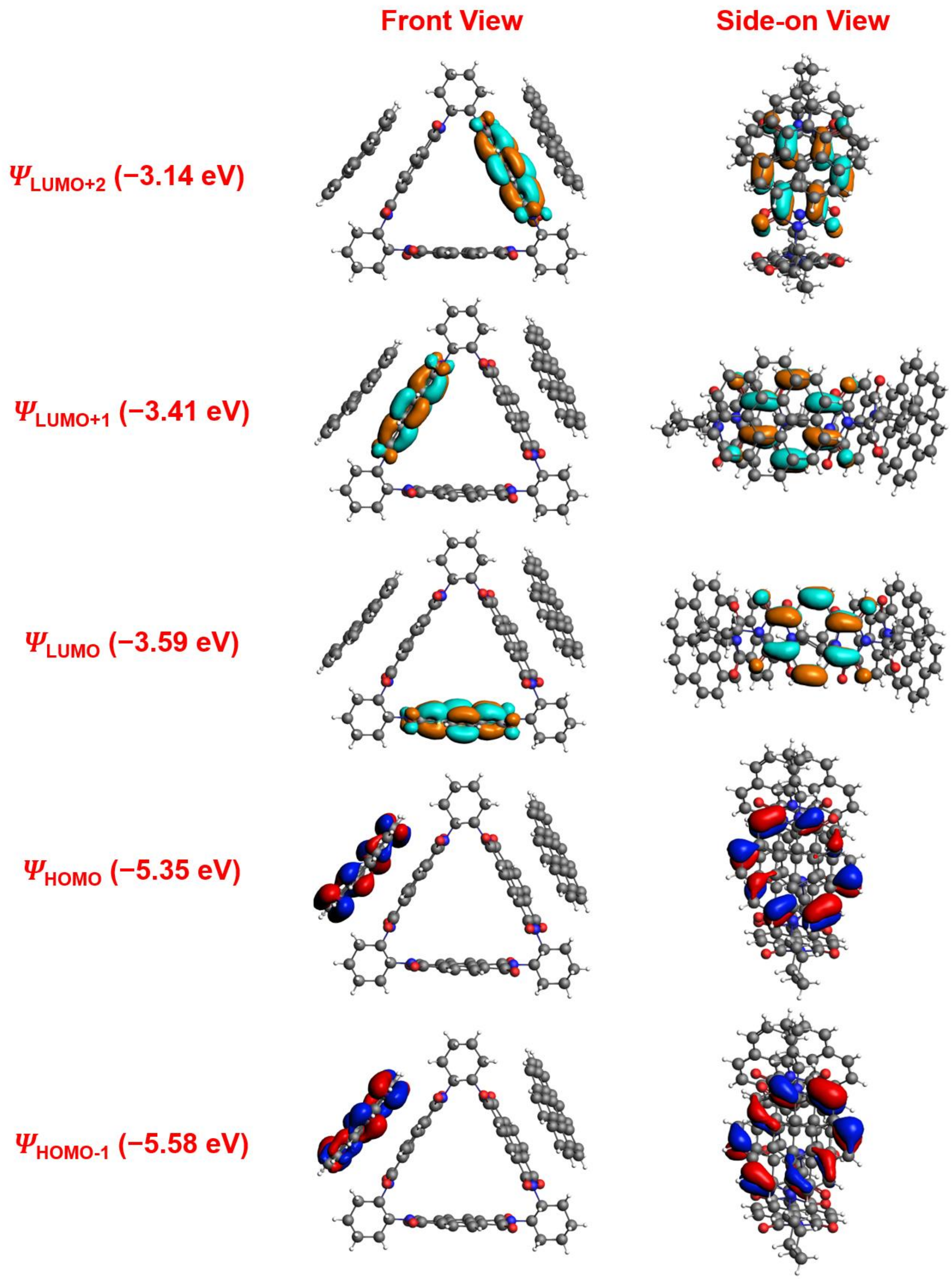

Supplementary Figure 34. Frontier molecular orbitals of **CNC-Q**. The energy bandgap is calculated to be 1.76 eV.

Supplementary Table 5. The Calculated Energy Levels of Frontier Molecular Orbitals

Energy / eV	NDI-Δ	CNC-T	CNC-Q	COR
Ψ_{LUMO+1}	−3.58	−3.57	−3.41	−1.65
Ψ_{LUMO}	−3.67	−3.64	−3.59	−1.72
Ψ_{HOMO}	−7.15	−5.64	−5.35	−5.68
Ψ_{HOMO-1}	−7.20	−6.02	−5.58	−5.75
$\Delta E_{HOMO\text{-}LUMO}$	3.47	2.00	1.76	3.97

(2) TDDFT-Calculated of One-Photon Absorption Spectra

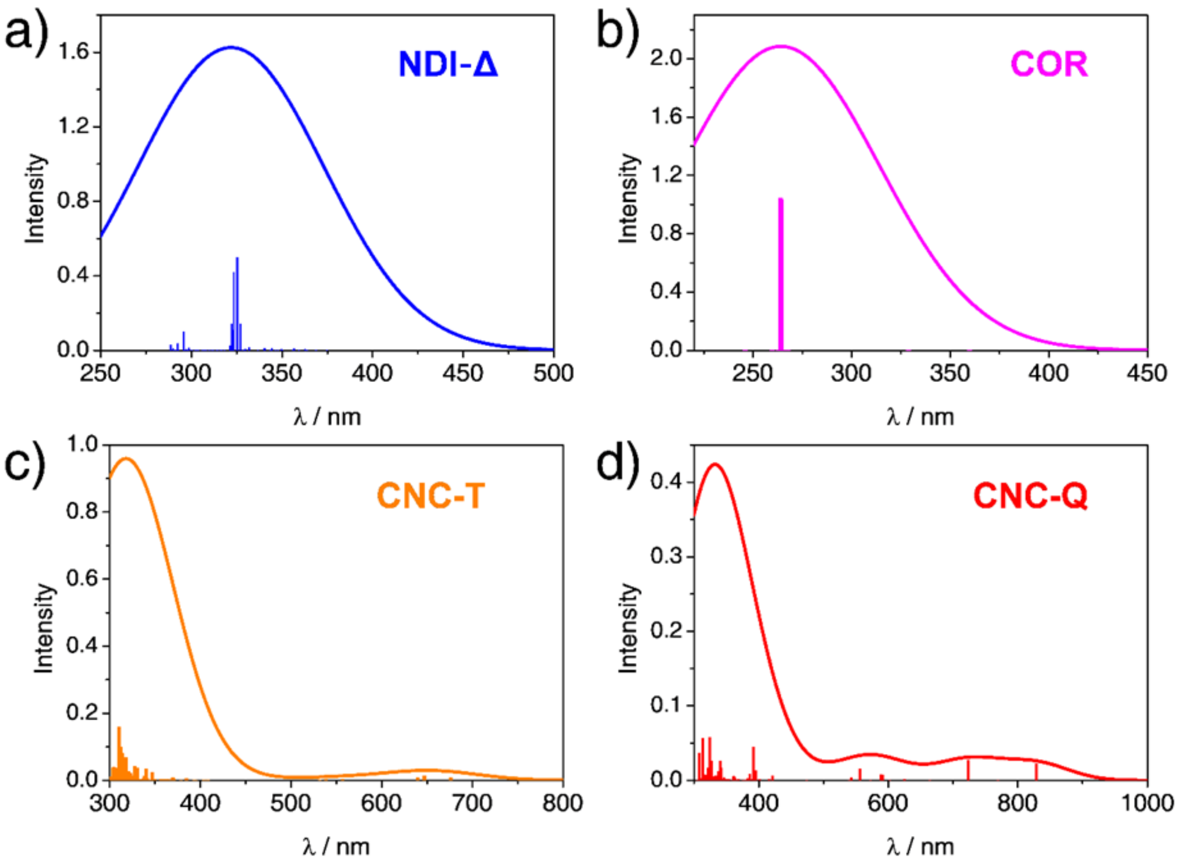

Supplementary Figure 35. Calculated one-photon absorption spectra of (a) **NDI-Δ**, (b) **COR**, (c) **CNC-T** and (d) **CNC-Q**

Supplementary Table 6. The Calculated Four Lowest Energy States of CNC-T and CNC-Q

CNC-T		CNC-Q	
**Wavelength / nm**	**Oscillator Strength / a.u.**	**Wavelength / nm**	**Oscillator Strength / a.u.**
677	0.009	827	0.020
646	0.014	770	0.001
639	0.007	725	0.027
556	0.004	663	0.001

Supplementary References

1. Dolomanov, O. V.; Bourhis, L. J.; Gildea, R. J.; Howard, J. A. K.; Puschmann, H. OLEX2: A complete structure solution, refinement and analysis program. *J. Appl. Cryst.* **42**, 339−341 (2009).
2. Sheldrick, G. M. SHELXT—integrated space-group and crystal-structure determination. *Acta. Cryst.* **A71**, 3−8 (2015).
3. Sheldrick, G. M. A Short history of SHELX. *Acta. Cryst.* **A64**, 112−122 (2008).
4. Thorn, A.; Dittrich, B.; Sheldrick, G. M. Enhanced rigid-bond restraints. *Acta. Cryst.* **A68**, 448−451 (2012).
